# Allele and haplotype frequencies of human leukocyte antigen-A, -B, -C, -DRB1, -DRB3/4/5, -DQA1, -DQB1, -DPA1, and -DPB1 by next generation sequencing-based typing in Koreans in South Korea

**DOI:** 10.1371/journal.pone.0253619

**Published:** 2021-06-21

**Authors:** In-Cheol Baek, Eun-Jeong Choi, Dong-Hwan Shin, Hyoung-Jae Kim, Haeyoun Choi, Tai-Gyu Kim

**Affiliations:** 1 Hematopoietic Stem Cell Bank, College of Medicine, The Catholic University of Korea, Seoul, South Korea; 2 Department of Microbiology, College of Medicine, The Catholic University of Korea, Seoul, South Korea; University of California San Francisco, UNITED STATES

## Abstract

Allele frequencies and haplotype frequencies of HLA-A, -B, -C, -DRB1, -DRB3/4/5, -DQA1, -DQB1, -DPA1, and -DPB1 have been rarely reported in South Koreans using unambiguous, phase-resolved next generation DNA sequencing. In this study, HLA typing of 11 loci in 173 healthy South Koreans were performed using next generation DNA sequencing with long-range PCR, TruSight^®^ HLA v2 kit, Illumina MiSeqDx platform system, and Assign^™^ for TruSight^™^ HLA software. Haplotype frequencies were calculated using the PyPop software. Direct counting methods were used to investigate the association with DRB1 for samples with only one copy of a particular secondary DRB locus. We compared these allele types with the ambiguous allele combinations of the IPD-IMGT/HLA database. We identified 20, 40, 26, 31, 19, 16, 4, and 16 alleles of HLA-A, HLA-B, HLA-C, HLA-DRB1, HLA-DQA1, HLA-DQB1, HLA-DPA1, and HLA-DPB1, respectively. The number of HLA-DRB3/4/5 alleles was 4, 5, and 3, respectively. The haplotype frequencies of most common haplotypes were as follows: A*33:03:01-B*44:03:01-C*14:03-DRB1*13:02:01-DQB1*06:04:01-DPB1*04:01:01 (2.89%), A*33:03:01-B*44:03:01-C*14:03 (4.91%), DRB1*08:03:02-DQA1*01:03:01-DQB1*06:01:01-DPA1*02:02:02-DPB1*05:01:01 (5.41%), DRB1*04:05:01-DRB4*01:03:01 (12.72%), DQA1*01:03:01-DQB1*06:01:01 (13.01%), and DPA1*02:02:02-DPB1*05:01:01 (30.83%). In samples with only one copy of a specific secondary DRB locus, we examined its association with DRB1. We, thus, resolved 10 allele ambiguities in HLA-B, -C (each exon 2+3), -DRB1, -DQB1, -DQA1, and -DPB1 (each exon 2) of the IPD-IMGT/HLA database. Korean population was geographically close to Japanese and Han Chinese populations in the genetic distances by multidimensional scaling (MDS) plots. The information obtained by HLA typing of the 11 extended loci by next generation sequencing may be useful for more exact diagnostic tests on various transplantations and the genetic population relationship studies in South Koreans.

## Introduction

It is widely known that human leukocyte antigen (HLA) matching reduces morbidity and mortality in patients after hematopoietic stem cell transplantation (HSCT) [1]. Traditionally, Sanger sequencing has been the standard method for high-resolution HLA typing (PCR-SBT) [2], as the likelihood of accepting HLA mismatches for sensitized organ transplant candidates should be determined at high resolution, not at the antigen level [3]. However, SBT is unable to accurately phase heterozygous alleles and only provides limited sequencing information. Typically, SBT protocols cover only the exons 2, 3, and 4 for HLA class I genes and exons 2 and 3 for class II genes. This problem of ambiguity, the presence of two or more genotypes compatible with the same unphased sequence generated by SBT, is evidence for the complexity of the HLA region in the human genome, which contains more than 28,000 alleles identified in the IPD-IMGT/HLA database [4]. Time-consuming and costly additional testing is needed, and the vague list of delaying patient outcomes is still growing.

In recent years, efforts have been made to resolve this ambiguity and to reduce time and money, using next generation sequencing (NGS) techniques [5]. High-resolution and high-throughput HLA typing methods have been validated using amplicon-based next generation DNA sequencing [6, 7], greatly simplifying the workflow [8]. We reported on the distributions of HLA-A, -B (exon 2 and 3) and -DRB1 (exon 2) using amplicon-based NGS in a previous study [9]. Furthermore, HLA typing using NGS has subsequently been performed by long-range amplification, sequencing platforms, and analysis algorithms [10–12]. These efforts decreased the ambiguity of HLA alleles for all 11 HLA loci [3, 13–19].

The distribution of HLA alleles differed significantly between ethnic groups, and specific alleles and haplotypes are characteristic of each ethnic group [9, 14, 16, 17, 20–32]. Transplant and disease-related studies, such as organ or hematopoietic stem cell transplantation, require data on the distribution of HLA alleles and haplotypes in each ethnic group [3, 33–35]. More extended HLA typing region is needed to improve the success rate of unrelated hematopoietic stem cell transplantation [36]. In this study, we analyzed the allele and haplotype frequencies of 11 extended HLA loci using long-range PCR.

## Methods

### Sample preparation

DNA was collected from the blood of 173 genetically unrelated healthy Korean adults who were mainly consistent with students and staff from the Medical College of the Catholic University of Korea in Seoul in South Korea. The Korean people are originally derived from one ethnic group, Mongolian who migrated to the Korean peninsula about five thousand years ago, and preserved the unique physico/anthropological characteristics. South Korea is rapidly developed and urbanization is accelerating by fast-paced industrialization after the Korean War between 1950 and 1953. These phenomenons make the huge population influx to Seoul from the different rural areas. In this way, Seoul became a metropolitan and features of special geographic origins have been diluted. Moreover, genetic homogeneity was revealed on the Korean peninsula level, except Jeju [37]. The population of the Seoul Capital Area (Seoul, Incheon, and Gyeonggi) amounted to 25.89 million persons in 2019, which accounted for 50.0% of the total population of South Korea. The 173 Korean adult from medical school, the subject of this study, has been used as a group representing Koreans because it includes diverse Koreans locally (http://kostat.go.kr/portal/eng/pressReleases/8/7/index.board?bmode=download&bSeq=&aSeq=386088&ord=1). Genomic DNA was freshly extracted from 4 mL of peripheral blood mixed with ethylenediaminetetraacetic acid (EDTA) using the TIANamp Genomic DNA Extraction Kits (Tiangen Biotech Corporation, Beijing, China), according to the manufacturer’s instructions. Extracted DNA was adjusted to a concentration of 50 ng/μL in Tris-ethylenediaminetetraacetic acid (TE) buffer [35], and DNA was quantified using a QuBit fluorometer (Life Technologies, Carlsbad, CA). After quantification, sample DNA was diluted to 10 ng/mL. All subjects provided informed consent to participate in genetic studies. Also, written informed consent was obtained from each participant. This research protocol was carried out in accordance with the Declaration of Helsinki with the approval of the Catholic University Institutional Review Board (IRB) (IRB number: MC13SISI0126).

### HLA gene amplification

Freshly extracted genomic DNA was used to amplify each HLA locus according to the manufacturer’s instructions: TruSight^®^ HLA v2 Sequencing Panel. PCR amplicons were confirmed using 2% agarose gel electrophoresis prior to preparing the NGS libraries. Twenty-four samples (192 HLA loci) were run in a single NGS experiment. The samples were of sufficient quality to ensure library preparation, data quality, and analysis, as well as the correct HLA typing by NGS.

### Genotyping of HLA alleles by Assign^™^ for TruSight^™^ HLA software

Data analysis was performed using Assign^™^ for TruSight^™^ HLA software (version 2.1.0.943, Illumina Inc., San Diego, CA). Sequencing data was interpreted on using the IPD-IMGT/HLA database 3.42.0 [4]. We compared the genotypes obtained with next-generation sequencing with the previous results acquired with Sanger sequencing, allowing the estimative of the NGS accuracy [35]. The Assign^™^ for TruSight^™^ HLA software was designed for the genotyping of HLA-A, -B, -C, -DRB1, -DRB3/4/5, -DQA1, -DQB1, -DPA1, and -DPB1 from the fastq sequencing reads provided by the Illumina MiSeqDx platform with the Illumina Pipeline software. The methods implemented within the Assign^™^ for TruSight^™^ HLA software utilizes a large number of reads per sequence.

### Statistical analysis

#### Allele and haplotype frequencies

The allele frequencies were determined using a direct counting method. Haplotypes were calculated using the iterative Expectation-Maximization (EM) algorithm [38, 39] implemented by the software PyPop-Win32-0.7.0 (www.pypop.org) for the HLA-A, -B, -C, -DRB1, -DQA1, -DQB1, -DPA1, and -DPB1 (S1–S5 Tables) [40]. The list of files that make up the PyPop-Win32-0.7.0 software contains a minimal configuration file called “sample.ini”. The presence of [Emhaplofreq] section in the file enables haplotype estimation. In ‘lociToEstHaplo’ option in the section you can list the multi-locus haplotypes for which you wish the program to estimate (S6 Table).

Genotyping data were further investigated for predicted haplotypes using the PyPop-Win32-0.7.0 software. This analysis was not performed on all 11 loci of the HLA gene due to the limitation in the number of samples in this study. The association of DRB1 with DRB3/4/5 was analyzed by direct counting.

#### Association of DRB1 with secondary DR loci (HLA-DRB3, -DRB4, and -DRB5)

Although not all samples present a secondary DR loci (HLA-DRB3, -DRB4, and -DRB5), direct counting methods were used to investigate association with DRB1 for samples with only one copy of a particular secondary DRB locus. The association of DRB1 with DRB3/4/5 loci were also investigated by comparison with previously reported DRB structures [41]. We referred to a two-field nomenclature found in volunteers from the US registry with European backgrounds and a prior population study of the Netherlands consistent with the more recent high-resolution haplotype assignments [14, 22, 23, 25, 26, 34, 42].

#### Hardy-Weinberg equilibrium

Eight loci (HLA-A, -B, -C, -DRB1, -DQA1, -DQB1, -DPA1, and -DPB1) were tested for Hardy-Weinberg equilibrium using SNPStats (https://www.snpstats.net/start.htm). The haplotypes including HLA-DRB1 and one of the non-classic HLA-DRB loci (HLA-DRB3, -DRB4, or -DRB5) were considered one locus for the HW test. If the P-value was less than 0.05, the locus was considered unbalanced.

#### Analysis of HLA alleles resolved on ambiguous allele combinations of the IPD-IMGT/HLA database (release version 3.42.0)

HLA alleles obtained from NGS by the TruSight^®^ HLA v2 kit were compared with the IPD/IMGT/HLA ambiguous allele combinations (exon 2+3 in HLA-A, -B, and -C and exon 2 in HLA-DRB1/3/4/5, -DQA1, -DQB1, -DPA1, and -DPB1) (S7 Table) [4].

#### Multidimensional scaling (MDS) analysis

MDS analysis (for two dimensions, based on the Euclidian distance matrix computed using allele frequencies) were performed with ALSCAL procedure using SPSS 27 software package (SPSS Inc., Chicago, IL, USA). ALSCAL procedure options were set to ‘interval’ for measurement level, ‘Symmetric’ for data matrix shape, ‘Dissimilarity’ for type, and ‘Leave Tied’ for Approach to Ties. For HLA-A, -B, -DRB1, -DQA1, and -DQB1, we analyzed using HLA data reported by Johansson et al [43]. For HLA-C, -DPA1, and -DPB1, HLA data collected from a worldwide selection of populations in Allelefriquencies.net database (http://www.allelefrequencies.net). The Japanese and Han Chinese populations were included because it was genetically close to this population in all analyzes, except for HLA-DPA1. Additionally, HLA data of deferent geographic regions was used in this study—HLA-C (13 populations): Japanese, Han Chinese, Australian, Southeast Asian, South Asian, West Asian, Oceanian, European, South American, North American, North African, and Sub-Saharan African; HLA-DPA1 (10 populations): Japanese, Southeast Asian, South Asian, Oceanian, European, Brazilian, South American, North American, and Sub-Saharan African; HLA-DPB1 (13 populations): Japanese, Han Chinese, Australian, Southeast Asian, South Asian, West Asian, Oceanian, European, South American, North American, North African, and Sub-Saharan African. The population groups were determined by ‘Region’ of HLA-Allele Frequency Search-Classical section in Allelefrequencies.net. Additionally, we selected to each country for ‘Country’ and ‘2 field’ for ‘Level of resolution’ (e.g. Han Chinese population was selected to ‘North-East Asia’ for ‘Region’, ‘China’ for ‘Country’, and ‘2 field’ for ‘Level of resolution’.). HLA allele frequency of each population group was recalculated by following formula of Allelefrequencies.net:

‘Allele Frequency: Total number of copies of the allele in the population sample (Alleles / 2n) in decimal format.’

The genetic distances of 8 HLA loci were analyzed by the 2nd field allele frequencies of this study and the estimated allele frequencies of the population groups.

## Results

### Hardy-Weinberg equilibrium

Hardy-Weinberg equilibrium tests were performed on the eight HLA loci. The statistical P value of observed, expected homozygotes and heterozygotes are given in S8 Table. The results showed that the P values at the loci were all more than 0.05. There were no detectable deviations at each of the eight loci from the Hardy-Weinberg equilibrium. P values greater than 0.05 indicate that the population is consistent with the Hardy-Weinberg equilibrium [44].

### Allele frequencies of HLA-A, -B, -C, -DRB1, -DQA1, -DQB1, -DPA1, and -DPB1

Allele frequencies of HLA-A, -B, -C, -DRB1, -DQA1, -DQB1, -DPA1, and -DPB1 are listed in Table 1 (>1%, Complete tables were showed in S9 and S10 Tables). For the HLA-A locus, the A2 group accounted for 35.5% of all the HLA-A alleles. In total, twenty distinct alleles were identified for the HLA-A locus. Of these, A*02:01:01 was the most common allele, followed by A*33:03:01, A*24:02:01, A*11:01:01, A*02:06:01, and A*02:07:01 (>5%). The HLA-B locus showed the greatest diversity, with forty identified alleles. Allele frequencies of B*15:01:01, B*54:01:01, B*46:01:01, B*44:03:01, B*58:01:01, and B*35:01:01 were over 5%. In HLA-C, twenty six alleles were identified; C*01:02:01 was the most common allele, followed by C*03:03:01, C*07:02:01, C*03:04:01, C*04:01:01, C*03:02:02, and C*14:03:01 (>5%). The allele frequencies of C*07:01:02 and C*07:06:01 were 0.29% and 2.31%, respectively. In the HLA-DRB1 locus, 31 alleles were identified. DRB1*08:03:02 was the most common allele, followed by DRB1*13:02:01, DRB1*04:05:01, DRB1*07:01:01, DRB1*15:01:01, DRB1*01:01:01, DRB1*09:01:02, and DRB1*04:06:01 (>5%). Interestingly, there were 19 alleles in HLA-DQA1 locus, making it very diverse. DQA1*01:02:01 were the most common allele, followed by DQA1*01:03:01, DQA1*03:01:01, DQA1*03:03:01, DQA1*01:04:01, DQA1*02:01:01, DQA1*01:01:01, DQA1*03:02:01, and DQA1*06:01:01 (>5%). Sixteen alleles for the HLA-DQB1 were identified. The sum of allele frequencies of DQB1*03:01:01 and DQB1*03:03:02 were 22.54%. This included three DQ6 alleles (DQB1*06:01:01, DQB1*06:02:01, and DQB1*06:04:01), DQB1*03:02:01, DQB1*04:01:01, DQB1*02:02:01, DQB1*05:01:01, and DQB1*05:03:01 (>5%). The DQB1*02:01:01 and DQB1*02:02:01 were 2.31% and 7.51%, respectively. Four alleles for the HLA-DPA1 locus were identified, consisting of DPA1*01:03:01, DPA1*02:02:02, DPA1*02:01:01, and DPA1*01:04 in descending order frequency. In the HLA-DPB1 locus, 16 alleles were identified. DPB1*05:01:01 and DPB1*02:01:02 were the most common, followed by DPB1*04:02:01, DPB1*04:01:01, DPB1*13:01:01, and DPB1*02:02:01 (>5%). The number of HLA-DRB3/4/5 alleles was 4, 5, and 3, respectively. Allele frequencies of DRB3*02:02:01, DRB3*01:01:02, DRB3*03:01:01, DRB3*03:01:03, DRB4*01:03:01, DRB4*01:03:02, DRB4*01:02, DRB4*01:01:01, DRB5*01:01:01, DRB5*01:02:01, and DRB5*02:02 were 32.12%, 16.36%, 16.36%, 6.67%, 51.52%, 8.48%, 2.42%, 0.61%, 0.61%, 13.94%, 6.67%, and 0.61%, respectively.

**Table 1:** Allele frequencies of HLA-A, -B, -C, -DRB1, -DQA1, -DQB1, -DPA1, and -DPB1 in South Koreans (N = 173, >1%).

HLA alleles	2n	%	HLA alleles	2n	%	HLA alleles	2n	%
A*02:01:01	79	22.83	C*03:02:02	19	5.49	DQA1*03:01:01	39	11.27
A*02:06:01	22	6.36	C*03:03:01	46	13.29	DQA1*03:02:01	19	5.49
A*02:07:01	18	5.20	C*03:04:01	32	9.25	DQA1*03:03:01	37	10.69
A*03:01:01	4	1.16	C*04:01:01	21	6.07	DQA1*05:01:01	11	3.18
A*11:01:01	45	13.01	C*05:01:01	4	1.16	DQA1*05:03:01	5	1.45
A*24:02:01	55	15.90	C*06:02:01	17	4.91	DQA1*05:05:01	16	4.62
A*26:01:01	14	4.05	C*07:02:01	39	11.27	DQA1*05:06:01	4	1.16
A*26:02:01	8	2.31	C*07:04:01	4	1.16	DQA1*05:08	7	2.02
A*30:01:01	10	2.89	C*07:06:01	8	2.31	DQA1*06:01:01	18	5.20
A*30:04:01	5	1.45	C*08:01:01	12	3.47			
A*31:01:02	12	3.47	C*08:03:01	6	1.73	DQB1*02:01:01	8	2.31
A*33:03:01	56	16.18	C*12:02:02	5	1.45	DQB1*02:02:01	26	7.51
			C*14:02:01	11	3.18	DQB1*03:01:01	57	16.47
B*07:02:01	17	4.91	C*14:03:01	19	5.49	DQB1*03:02:01	41	11.85
B*13:01:01	11	3.18	C*15:02:01	9	2.60	DQB1*03:03:02	21	6.07
B*13:02:01	17	4.91				DQB1*04:01:01	29	8.38
B*15:01:01	38	10.98	DRB1*01:01:01	20	5.78	DQB1*04:02:01	6	1.73
B*15:07:01	4	1.16	DRB1*03:01:01	8	2.31	DQB1*05:01:01	23	6.65
B*15:11:01	5	1.45	DRB1*04:01:01	4	1.16	DQB1*05:02:01	12	3.47
B*15:18:01	4	1.16	DRB1*04:03:01	12	3.47	DQB1*05:03:01	18	5.20
B*27:05:02	11	3.18	DRB1*04:05:01	29	8.38	DQB1*06:01:01	46	13.29
B*35:01:01	18	5.20	DRB1*04:06:01	18	5.20	DQB1*06:02:01	22	6.36
B*38:02:01	5	1.45	DRB1*07:01:01	27	7.80	DQB1*06:03:01	4	1.16
B*40:01:02	11	3.18	DRB1*08:02:01	9	2.60	DQB1*06:04:01	21	6.07
B*40:02:01	15	4.34	DRB1*08:03:02	40	11.56	DQB1*06:09:01	11	3.18
B*40:06:01	7	2.02	DRB1*09:01:02	19	5.49			
B*44:02:01	4	1.16	DRB1*11:01:01	14	4.05	DPA1*01:03:01	152	43.93
B*44:03:01	20	5.78	DRB1*12:01:01	17	4.91	DPA1*02:01:01	54	15.61
B*44:03:02	9	2.60	DRB1*12:02:01	12	3.47	DPA1*02:02:02	139	40.17
B*46:01:01	21	6.07	DRB1*13:01:01	4	1.16			
B*48:01:01	13	3.76	DRB1*13:02:01	32	9.25	DPB1*02:01:02	87	25.14
B*51:01:01	17	4.91	DRB1*14:05:01	13	3.76	DPB1*02:02:01	18	5.20
B*51:02:01	4	1.16	DRB1*14:54:01	12	3.47	DPB1*03:01:01	16	4.62
B*52:01:01	5	1.45	DRB1*15:01:01	23	6.65	DPB1*04:01:01	21	6.07
B*54:01:01	27	7.80	DRB1*15:02:01	12	3.47	DPB1*04:02:01	30	8.67
B*55:02:01	6	1.73				DPB1*05:01:01	118	34.10
B*58:01:01	20	5.78	DQA1*01:01:01	23	6.65	DPB1*09:01:01	11	3.18
B*59:01:01	10	2.89	DQA1*01:02:01	55	15.90	DPB1*13:01:01	20	5.78
			DQA1*01:03:01	49	14.16	DPB1*14:01:01	8	2.31
C*01:02:01	72	20.81	DQA1*01:04:01	27	7.80	DPB1*17:01:01	11	3.18
C*02:02:02	4	1.16	DQA1*02:01:01	27	7.80			

### Haplotype analysis

The 6-locus haplotypes of HLA-A, -B, -C, -DRB1, -DQB1, and -DPB1 are listed in Table 2. Haplotype frequencies of 14 HLA-A, -B, -C, -DRB1, -DQB1, and -DPB1 were >1%. A*33:03:01-B*44:03:01-C*14:03-DRB1*13:02:01-DQB1*06:04:01-DPB1*04:01:01 (2.89%) was the most common haplotype.

**Table 2:** Haplotype frequencies of HLA-A, -B, -C, -DRB1, -DQB1, and -DPB1 (>1%).

HLA haplotypes	HF (%)
A*33:03:01-B*44:03:01-C*14:03-DRB1*13:02:01-DQB1*06:04:01-DPB1*04:01:01	2.89
A*02:07:01-B*46:01:01-C*01:02:01-DRB1*08:03:02-DQB1*06:01:01-DPB1*05:01:01	2.60
A*33:03:01-B*44:03:02-C*07:06-DRB1*07:01:01-DQB1*02:02:01-DPB1*13:01:01	1.73
A*30:01:01-B*13:02:01-C*06:02:01-DRB1*07:01:01-DQB1*02:02:01-DPB1*17:01:01	1.73
A*02:01:01-B*54:01:01-C*01:02:01-DRB1*04:05:01-DQB1*04:01:01-DPB1*05:01:01	1.73
A*24:02:01-B*07:02:01-C*07:02:01-DRB1*01:01:01-DQB1*05:01:01-DPB1*04:02:01	1.73
A*33:03:01-B*44:03:01-C*14:03-DRB1*13:02:01-DQB1*06:04:01-DPB1*02:01:02	1.45
A*02:01:01-B*15:01:01-C*03:03:01-DRB1*12:01:01-DQB1*03:01:01-DPB1*02:01:02	1.45
A*02:01:01-B*15:01:01-C*04:01:01-DRB1*04:06:01-DQB1*03:02:01-DPB1*05:01:01	1.45
A*24:02:01-B*07:02:01-C*07:02:01-DRB1*01:01:01-DQB1*05:01:01-DPB1*05:01:01	1.16
A*24:02:01-B*54:01:01-C*01:02:01-DRB1*04:05:01-DQB1*04:01:01-DPB1*05:01:01	1.16
A*11:01:01-B*15:01:01-C*04:01:01-DRB1*04:06:01-DQB1*03:02:01-DPB1*02:01:02	1.16
A*33:03:01-B*58:01:01-C*03:02:02-DRB1*13:02:01-DQB1*06:09:01-DPB1*05:01:01	1.16
A*24:02:01-B*52:01:01-C*12:02:02-DRB1*15:02:01-DQB1*06:01:01-DPB1*09:01:01	1.16

HF, haplotype frequency

The haplotype frequencies of HLA class I genes, HLA-A, -B, and -C, with values >1% are displayed in S11 Table except for overlapping parts of Table 2. The remaining eight kinds were A*11:01:01-B*54:01:01-C*01:02:01 (2.49%), A*24:02:01-B*59:01:01-C*01:02:01 (2.31%), A*02:01:01-B*40:02:01-C*03:04:01 (2.02%), A*02:01:01-B*13:01:01-C*03:04:01 (1.45%), A*02:01:01-B*15:11:01-C*03:03:01 (1.45%), A*02:01:01-B*27:05:02-C*01:02:01 (1.44%), A*11:01:01-B*35:01:01-C*03:03:01 (1.13%), and A*02:06:01-B*46:01:01-C*01:02:01 (1.12%). The sum of the top ten haplotype frequencies was approximately 32.15%.

The frequencies of the 24 haplotypes of HLA class II genes, HLA-DRB1, -DQA1, -DQB1, -DPA1 and -DPB1, are shown in S12 Table (>1%). DRB1*08:03:02-DQA1*01:03:01-DQB1*06:01:01-DPA1*02:02:02-DPB1*05:01:01 (5.41%) was the most common allele. The haplotype frequencies of 2-locus haplotypes of HLA class II genes are shown in S13 Table (>1%). 19 out of the 25 observed haplotypes including HLA-DRB1 and one of the non-classic HLA-DRB loci (HLA-DRB3, -DRB4, or -DRB5) presented frequencies over 1%. In samples with only one copy of a specific secondary DRB locus, we examined the association with DRB1. The DRB1*04:05:01-DRB4*01:03:01 haplotype had the highest frequency (22/173, 12.72%), followed by DRB1*13:02:01-DRB3*03:01:01 (16/173, 9.25%), DRB1*04:06:01-DRB4*01:03:01 (14/173, 8.09%), DRB1*07:01:01-DRB4*01:03:01 (11/173, 6.36%), DRB1*15:01:01-DRB5*01:01:01 (11/173, 6.36%), DRB1*09:01:02-DRB4*01:03:02 (10/173, 5.78%), DRB1*15:02:01-DRB5*01:02 (10/173, 5.78%), DRB1*12:01:01-DRB3*02:02:01 (9/173, 5.20%), and DRB1*14:54:01-DRB3*02:02:01 (9/173, 5.20%) (>5%). Twenty DQA1~DQB1 and fifteen DPA1~DPB1 haplotypes presented frequencies over 1%, wherein DQA1*01:03:01-DQB1*06:01:01 (13.01%) and DPA1*02:02:02-DPB1*05:01:01 (30.83%) were the most common haplotypes, respectively.

### Expected PCR-SBT ambiguities (described in the IPD-IMGT/HLA database) solved by the NGS assay

Analysis of the data obtained from NGS by the TruSight^®^ HLA v2 kit and Assign^™^ for TruSight^™^ HLA software resolved 10 allele ambiguities in HLA-B, -C (each exon 2+3), -DRB1, -DQB1, -DQA1, and -DPB1 (each exon 2) of the IPD-IMGT/HLA database (Release version 3.42.0) (S14 Table). The unresolved ambiguities observed using the PCR-sequence based typing method were in the IPD-IMGT/HLA database.

### Genetic distances by MDS plots

**Fig 1:**
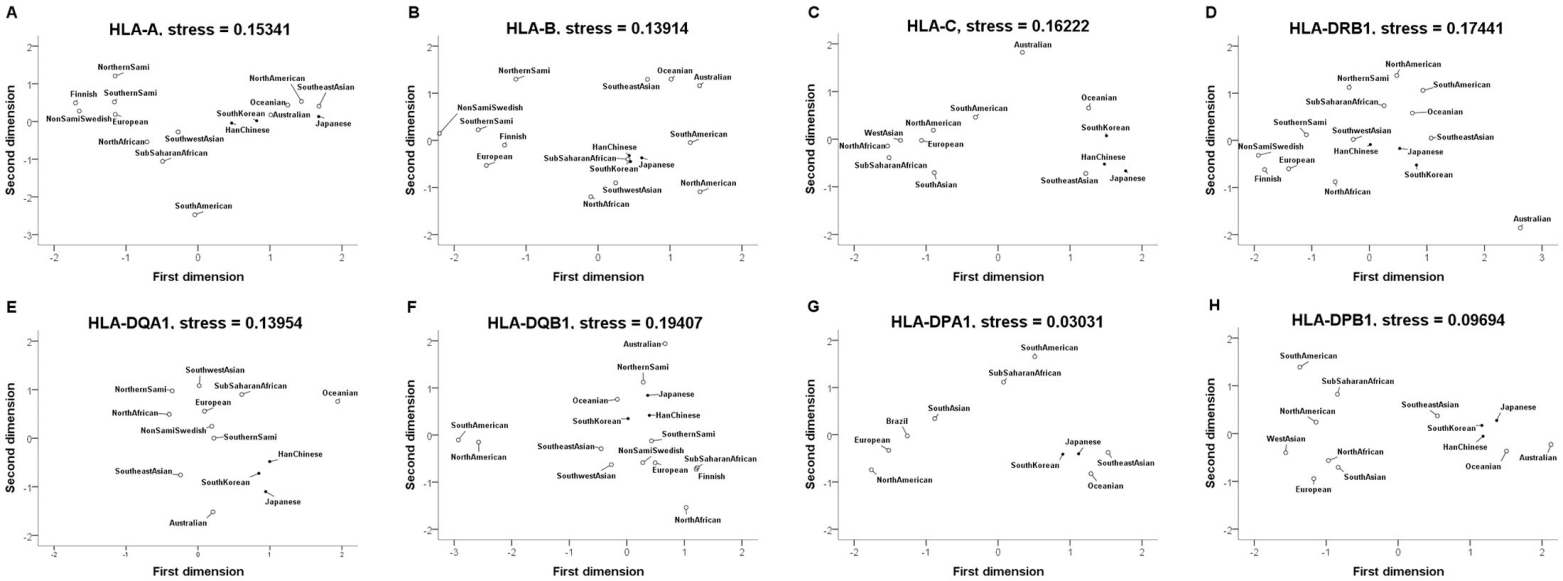
Euclidian distances by two dimensional MDS plots for allele frequencies of 8 HLA loci. (A) HLA-A, (B) HLA-B, (C) HLA-C, (D) HLA-DRB1, (E) HLA-DQA1, (F) HLA-DQB1, (G) HLA-DPA1, (H) HLA-DPB1.

According to (1) the 2nd field allele frequencies of this study, (2) the estimated allele frequencies of the population groups, and (3) the allele frequencies reported by Johansson et al [43], allele frequencies of HLA-A, -B, -C, -DRB1, -DQA1, -DQB1, -DPA1, and -DPB1 in 10–16 populations were collected to analyze Euclidian genetic distances by MDS plots (S15–S22 Tables). Since there is no data on HLA-DPA1 in some ethnicities, genetic distances were calculated only for 10 populations. The MDS analysis resulted in stress values of 0.15341, 0.13914, 0.16222, 0.17441, 0.13954, 0.19407, 0.03031, and 0.09694 for HLA-A, -B, -C, -DRB1, -DQA1, -DQB1, -DPA1, and -DPB1 data, respectively. Interestingly, the stress values in HLA-DPA1 and -DPB1 were Excellent and Perfect by Kruskal’s stress formula 1, respectively. In all analyzed genes, Korean, Japanese, and Han Chinese populations included in Northeast Asian populations were found to be close ethnicity, except for HLA-DPA1. In the analysis of HLA-DPB1, Northeast Asian was relatively close to Oceanian, Southeast Asian, and Australian populations compared to other ethnicities (Fig 1).

## Discussion

We first reported all the 11 loci HLA typing in South Koreans. In order to obtain high-resolution HLA types (3rd field allele types), 173 healthy Koreans were the HLA typed for all 11 HLA loci using next generation DNA sequencing (HLA-A, -B, -C, -DRB1, -DRB3/4/5, -DQA1, -DQB1, -DPA1 and -DPB1), of which were 100% concordant across HLA-A, -B, and -DRB1 (to 3rd field) [9]. No historical data was present for 3rd field of HLA-C, -DRB3/4/5, -DQA1, -DQB1, -DPA1, and -DPB1 (Table 1).

Compared with previous studies [9, 23, 24, 27, 30], the HLA-C, -DRB3/4/5, -DQA1, -DQB1, -DPA1, and -DPB1 alleles that were determined at high resolution in this study could be helpful for a clinical decision. The tolerance of a mismatch will depend on the patient’s clinic and the discrepancy between unmatched alleles. We compared the allele types of 11 HLA loci in the Netherlands, UK, Hong Kong, Chinese, Philippines, and Vietnamese [14, 16, 17, 29, 31, 32]. A*26:02 and B*15:27 are already known alleles that are not uncommon in South Koreans and Japanese, and B*55:07 is also found infrequently since it was reported as a variant of the B55 serotype in 1999 [45]. Some of the identified B*54:19, C*03:02:01, C*08:06, C*14:39, DRB1*11:45, DRB1*13:198, DRB1*14:06:01, and DRB1*14:142 alleles are included in the CWD list (CWD catalogue version 2.0, CIWD version 3.0.0) [46, 47], and some are rare ones. It is necessary to make a distinction between the two. Comparison of the Japanese populations (2nd field, 6 loci) [28] that were most close to the South Koreans, the alleles were B*54:19, B*55:07, C*08:06, C*14:39, DRB1*11:45, DRB1*13:198, and DRB1*14:142. When creating commercial kits for South Koreans, manufacturers and developers must add these unique alleles to the test subject. When performing the HLA typing before transplantation for donors and patients, researchers will also need to include these unique alleles in the test so that they can be detected.

Compared with the Japanese population, in the 6 HLA loci [20], 7 kinds of the haplotypes with frequencies higher than 1% have been found in both populations, which were A*02:07:01-B*46:01:01-C*01:02:01-DRB1*08:03:02-DQB1*06:01:01-DPB1*05:01:01, A*02:01:01-B*15:01:01-C*03:03:01-DRB1*12:01:01-DQB1*03:01:01-DPB1*02:01:02, A*33:03:01-B*44:03:01-C*14:03-DRB1*13:02:01-DQB1*06:04:01-DPB1*04:01:01, A*24:02:01-B*07:02:01-C*07:02:01-DRB1*01:01:01-DQB1*05:01:01-DPB1*05:01:01, A*11:01:01-B*15:01:01-C*04:01:01-DRB1*04:06:01-DQB1*03:02:01-DPB1*02:01:02, A*24:02:01-B*52:01:01-C*12:02:02-DRB1*15:02:01-DQB1*06:01:01-DPB1*09:01:01, and A*24:02:01-B*07:02:01-C*07:02:01-DRB1*01:01:01-DQB1*05:01:01-DPB1*04:02:01. The haplotype frequency of A*33:03:01-B*44:03:02-C*07:06-DRB1*07:01:01-DQB1*02:02:01-DPB1*13:01:01 (1.73%) in Table 2 was lower than that of the top ten kinds of South Asian (2.17%) [16]. The top 10 haplotype frequencies of HLA-A, -B, and -C were the same at the 2nd field in the previous study (S11 Table) [24]. In the HLA class II, the haplotypes of the 3rd field of HLA-DRB1, -DQA1, -DQB1, -DPA1, and -DPB1 (>1%) were not the same as the top ten most frequent haplotypes of different ethnic groups, European, South Asian, and African or Caribbean Black (S12 Table) [16]. Twenty-five DRB1-DRB3/4/5 haplotypes in S13 Table were investigated from the DRB1 and DRB3/4/5 genotyping data by comparison to the previously reported DRB structures [41, 42]. Only samples containing one copy of the secondary locus could also be evaluated against the previous study [14]. HLA genotyping data using the long-range PCR based NGS method are needed to understand the detailed DR haplotype structure and polymorphic generation. We compared the haplotype frequencies of 2-locus haplotypes of HLA class II genes in S13 Table, HLA-DQA1-DQB1 and HLA-DPA1-DPB1 (>1%) with those in Fukuoka, Japan [21]. The haplotypes shared 8 for HLA-DQA1-DQB1 and 9 for HLA-DPA1-DPB1, respectively.

The PCR-sequence based typing method was observed as unresolved ambiguities in the IPD-IMGT/HLA database (Release version 3.42.0) (S14 Table). To increase the success rate of solid organ or hematopoietic stem cell transplantation, more extensive and high-resolution HLA typing is required. For the selection of solid organ donors in hypersensitized patients, a change in HLA type is needed, recognizing the need for high-resolution HLA types in traditional serologically defined HLA antigens [3]. More extended HLA typing region is needed to improve the success rate of unrelated hematopoietic stem cell transplantation [36].

In mitochondrial DNA study, MDS plot and unrooted phylogenetic tree were no significant differences [37]. The collected samples are composed from the same ethnic group in South Korea and can be said to have genetic homogeneity. Compared to other ethnic groups, we were able to account for genetic distances with MDS plots (Fig 1). It highlights the need to use multiple loci to study genetic population relationships such as genetic distance of HLA genes [43, 48]. The genetic distance of South Koreans was measured for more extended HLA loci. The genetic distances of the HLA-A, -B, -C, -DRB1, -DQA1, -DQB1, -DPA1, and -DPB1 loci in the characteristics of the Korean samples support the theory that Koreans are primarily Northeast Asian origin. Our MDS analyses both genetic distance the South Koreans in close vicinity to Japanese and Han Chinese populations, whereas some analyses indicate a similarity to other Northeast Asian populations.

In conclusion, we analyzed the allele and haplotype frequencies of 11 entire and extensive HLA loci and the genetic distances by MDS plots compared to other ethnic groups. These data may be useful for more exact diagnostic tests of various transplantation and the genetic population relationship studies.

## Supporting information

S1 Table HLA-A, -B, and -DRB1 haplotype frequencies (>0.5%). (DOCX)

S2 Table HLA-A, -B, -C, and -DRB1 haplotype frequencies (>0.5%). (DOCX)

S3 Table HLA-A, -B, -C, -DRB1, and -DQB1 haplotype frequencies (>0.5%). (DOCX)

S4 Table HLA-A, -B, -C, -DRB1, -DQA1, and -DQB1 haplotype frequencies (>0.5%). (DOCX)

S5 Table HLA-DRB1, -DQB1, and -DPB1 haplotype frequencies (>0.5%). (DOCX)

S6 Table The entire configuration file (.ini). (DOCX)

S7 Table Alleles resolved on ambiguous allele combinations of the IPD-IMGT/HLA database (Release version 3.42.0) (n = 173). (DOCX)

S8 Table The Hardy-Weinberg equilibrium of HLA-A, -B, -C, -DRB1, -DQA1, -DQB1, -DPA1, and -DPB1 loci in South Korea population. (DOCX)

S9 Table Allele frequencies of HLA-A, -B, and -C in South Koreans (N = 173). (DOCX)

S10 Table Allele frequencies of HLA-DRB1, -DQA1, -DQB1, -DPA1, and -DPB1 in South Koreans (N = 173). (DOCX)

S11 Table Haplotype frequencies of HLA-A, -B, and -C except for overlapping parts of Table 2 (>1%). (DOCX)

S12 Table Haplotype frequencies of HLA-DRB1, -DQA1, -DQB1, -DPA1, and -DPB1 (>1%). (DOCX)

S13 Table Haplotype frequencies of 2-locus haplotypes of HLA class II genes (>1%). (DOCX)

S14 Table Expected PCR-SBT ambiguities (described in the IPD-IMGT/HLA database) solved by the NGS assay (n = 173). (DOCX)

S15 Table HLA-A allele frequencies of 16 populations. (DOCX)

S16 Table HLA-B allele frequencies of 16 populations. (DOCX)

S17 Table HLA-C allele frequencies of 13 populations. (DOCX)

S18 Table HLA-DRB1 allele frequencies of 16 populations. (DOCX)

S19 Table HLA-DQA1 allele frequencies of 15 populations. (DOCX)

S20 Table HLA-DQB1 allele frequencies of 16 populations. (DOCX)

S21 Table HLA-DPA1 allele frequencies of 10 populations. (DOCX)

S22 Table HLA-DPB1 allele frequencies of 14 populations. (DOCX)

## Abbreviations

HLA: Human leukocyte antigen
MDS: Multidimensional scaling
NGS: Next generation sequencing
