# Supplementary material for: Allele and haplotype frequencies of human leukocyte antigen-A, -B, -C, -DRB1, -DRB3/4/5, -DQA1, -DQB1, -DPA1, and -DPB1 by next generation sequencing-based typing in Koreans in South Korea
Source: PLoS One. 2021 Jun 21;16(6):e0253619. doi: 10.1371/journal.pone.0253619 (PMC8216545; doi:10.1371/journal.pone.0253619)
Supplement: S1 Table — (DOCX) [file pone.0253619.s001.docx]

**S1 Table**. HLA-A, -B, and -DRB1 haplotype frequencies (>0.5%)

| HLA haplotypes | HF (%) |
| --- | --- |
| A*33:03:01-B*44:03:01-DRB1*13:02:01 | 4.62 |
| A*33:03:01-B*58:01:01-DRB1*13:02:01 | 3.18 |
| A*24:02:01-B*07:02:01-DRB1*01:01:01 | 3.18 |
| A*02:07:01-B*46:01:01-DRB1*08:03:02 | 2.89 |
| A*30:01:01-B*13:02:01-DRB1*07:01:01 | 2.31 |
| A*11:01:01-B*15:01:01-DRB1*04:06:01 | 2.02 |
| A*33:03:01-B*44:03:02-DRB1*07:01:01 | 2.02 |
| A*02:01:01-B*54:01:01-DRB1*04:05:01 | 1.73 |
| A*02:01:01-B*15:01:01-DRB1*04:06:01 | 1.45 |
| A*02:01:01-B*13:01:01-DRB1*12:02:01 | 1.45 |
| A*24:02:01-B*59:01:01-DRB1*04:05:01 | 1.30 |
| A*02:07:01-B*46:01:01-DRB1*09:01:02 | 1.16 |
| A*02:01:01-B*15:01:01-DRB1*12:01:01 | 1.16 |
| A*24:02:01-B*54:01:01-DRB1*04:05:01 | 1.16 |
| A*24:02:01-B*52:01:01-DRB1*15:02:01 | 1.16 |
| A*02:01:01-B*27:05:02-DRB1*01:01:01 | 1.16 |
| A*02:01:01-B*15:01:01-DRB1*15:01:01 | 1.16 |
| A*26:01:01-B*40:02:01-DRB1*15:01:01 | 0.87 |
| A*02:01:01-B*40:02:01-DRB1*08:03:02 | 0.87 |
| A*02:01:01-B*54:01:01-DRB1*08:03:02 | 0.87 |
| A*29:01:01-B*07:05:01-DRB1*08:03:02 | 0.87 |
| A*33:03:01-B*58:01:01-DRB1*03:01:01 | 0.87 |
| A*24:02:01-B*13:02:01-DRB1*07:01:01 | 0.87 |
| A*03:02:01-B*08:01:01-DRB1*03:01:01 | 0.87 |
| A*11:01:01-B*13:01:01-DRB1*12:02:01 | 0.87 |
| A*02:01:01-B*40:02:01-DRB1*12:01:01 | 0.58 |
| A*02:01:01-B*13:01:01-DRB1*08:03:02 | 0.58 |
| A*02:01:01-B*54:01:01-DRB1*14:54:01 | 0.58 |
| A*02:01:01-B*35:01:01-DRB1*14:03:01 | 0.58 |
| A*24:02:01-B*51:01:01-DRB1*11:01:01 | 0.58 |
| A*11:01:01-B*15:01:01-DRB1*08:03:02 | 0.58 |
| A*24:02:01-B*48:01:01-DRB1*08:03:02 | 0.58 |
| A*02:06:01-B*40:03-DRB1*12:01:01 | 0.58 |
| A*30:04:01-B*35:01:01-DRB1*11:01:01 | 0.58 |
| A*02:06:01-B*54:01:01-DRB1*04:05:01 | 0.58 |
| A*11:01:01-B*54:01:01-DRB1*07:01:01 | 0.58 |
| A*11:01:01-B*44:02:01-DRB1*13:01:01 | 0.58 |
| A*11:01:01-B*15:07:01-DRB1*04:03:01 | 0.58 |
| A*31:01:02-B*07:02:01-DRB1*01:01:01 | 0.58 |
| A*24:02:01-B*59:01:01-DRB1*14:05:01 | 0.58 |
| A*24:02:01-B*51:01:01-DRB1*12:01:01 | 0.58 |
| A*26:02:01-B*15:01:01-DRB1*14:06:01 | 0.58 |
| A*31:01:02-B*51:01:01-DRB1*04:03:01 | 0.58 |
| A*02:06:01-B*40:02:01-DRB1*08:02:01 | 0.58 |
| A*33:03:01-B*40:06:01-DRB1*09:01:02 | 0.58 |
| A*02:01:01-B*40:01:02-DRB1*08:02:01 | 0.58 |
| A*33:03:01-B*14:01:01-DRB1*08:02:01 | 0.58 |
| A*02:06:01-B*46:01:01-DRB1*15:02:01 | 0.58 |
| A*02:03:01-B*38:02:01-DRB1*14:54:01 | 0.58 |
| A*26:01:01-B*48:01:01-DRB1*15:01:01 | 0.58 |
| A*11:01:01-B*51:01:01-DRB1*04:06:01 | 0.58 |
| A*02:01:01-B*15:18:01-DRB1*04:05:01 | 0.58 |

HF, haplotype frequency
