# Supplementary material for: Allele and haplotype frequencies of human leukocyte antigen-A, -B, -C, -DRB1, -DRB3/4/5, -DQA1, -DQB1, -DPA1, and -DPB1 by next generation sequencing-based typing in Koreans in South Korea
Source: PLoS One. 2021 Jun 21;16(6):e0253619. doi: 10.1371/journal.pone.0253619 (PMC8216545; doi:10.1371/journal.pone.0253619)
Supplement: S5 Table — (DOCX) [file pone.0253619.s005.docx]

**S5 Table**. HLA-DRB1, -DQB1, and -DPB1 haplotype frequencies (>0.5%)

| HLA haplotypes | HF (%) |
| --- | --- |
| DRB1*08:03:02-DQB1*06:01:01-DPB1*05:01:01 | 5.32 |
| DRB1*04:05:01-DQB1*04:01:01-DPB1*05:01:01 | 4.92 |
| DRB1*13:02:01-DQB1*06:04:01-DPB1*04:01:01 | 4.05 |
| DRB1*09:01:02-DQB1*03:03:02-DPB1*05:01:01 | 3.49 |
| DRB1*15:01:01-DQB1*06:02:01-DPB1*02:01:02 | 3.09 |
| DRB1*01:01:01-DQB1*05:01:01-DPB1*05:01:01 | 3.00 |
| DRB1*07:01:01-DQB1*02:02:01-DPB1*17:01:01 | 2.89 |
| DRB1*12:02:01-DQB1*03:01:01-DPB1*05:01:01 | 2.88 |
| DRB1*15:02:01-DQB1*06:01:01-DPB1*09:01:01 | 2.60 |
| DRB1*04:06:01-DQB1*03:02:01-DPB1*05:01:01 | 2.58 |
| DRB1*12:01:01-DQB1*03:01:01-DPB1*02:01:02 | 2.50 |
| DRB1*01:01:01-DQB1*05:01:01-DPB1*04:02:01 | 2.49 |
| DRB1*15:01:01-DQB1*06:02:01-DPB1*05:01:01 | 2.18 |
| DRB1*14:05:01-DQB1*05:03:01-DPB1*05:01:01 | 2.13 |
| DRB1*11:01:01-DQB1*03:01:01-DPB1*02:01:02 | 2.02 |
| DRB1*07:01:01-DQB1*02:02:01-DPB1*13:01:01 | 2.00 |
| DRB1*08:03:02-DQB1*06:01:01-DPB1*02:01:02 | 1.75 |
| DRB1*08:02:01-DQB1*03:02:01-DPB1*05:01:01 | 1.73 |
| DRB1*08:03:02-DQB1*06:01:01-DPB1*02:02 | 1.73 |
| DRB1*14:05:01-DQB1*05:03:01-DPB1*02:01:02 | 1.62 |
| DRB1*04:03:01-DQB1*03:02:01-DPB1*02:01:02 | 1.59 |
| DRB1*13:02:01-DQB1*06:09:01-DPB1*02:01:02 | 1.45 |
| DRB1*07:01:01-DQB1*02:02:01-DPB1*05:01:01 | 1.18 |
| DRB1*04:06:01-DQB1*03:02:01-DPB1*02:01:02 | 1.16 |
| DRB1*03:01:01-DQB1*02:01:01-DPB1*04:01:01 | 1.16 |
| DRB1*04:05:01-DQB1*04:01:01-DPB1*04:02:01 | 1.13 |
| DRB1*04:03:01-DQB1*03:02:01-DPB1*05:01:01 | 1.01 |
| DRB1*09:01:02-DQB1*03:03:02-DPB1*04:02:01 | 0.94 |
| DRB1*04:05:01-DQB1*04:01:01-DPB1*02:01:02 | 0.89 |
| DRB1*13:02:01-DQB1*06:04:01-DPB1*13:01:01 | 0.87 |
| DRB1*14:54:01-DQB1*05:02:01-DPB1*04:02:01 | 0.87 |
| DRB1*04:03:01-DQB1*03:02:01-DPB1*04:02:01 | 0.87 |
| DRB1*08:03:02-DQB1*03:01:01-DPB1*02:01:02 | 0.87 |
| DRB1*12:01:01-DQB1*03:01:01-DPB1*03:01:01 | 0.87 |
| DRB1*08:03:02-DQB1*03:01:01-DPB1*14:01:01 | 0.87 |
| DRB1*03:01:01-DQB1*02:01:01-DPB1*03:01:01 | 0.87 |
| DRB1*04:05:01-DQB1*04:01:01-DPB1*03:01:01 | 0.87 |
| DRB1*11:01:01-DQB1*03:01:01-DPB1*05:01:01 | 0.87 |
| DRB1*14:54:01-DQB1*05:02:01-DPB1*02:02 | 0.82 |
| DRB1*09:01:02-DQB1*03:03:02-DPB1*02:01:02 | 0.78 |
| DRB1*13:02:01-DQB1*06:04:01-DPB1*02:01:02 | 0.74 |
| DRB1*08:03:02-DQB1*06:01:01-DPB1*04:02:01 | 0.73 |
| DRB1*12:01:01-DQB1*03:01:01-DPB1*05:01:01 | 0.66 |
| DRB1*15:01:01-DQB1*06:02:01-DPB1*02:02 | 0.62 |
| DRB1*12:02:01-DQB1*03:01:01-DPB1*02:01:02 | 0.59 |
| DRB1*04:06:01-DQB1*03:02:01-DPB1*14:01:01 | 0.58 |
| DRB1*13:02:01-DQB1*06:09:01-DPB1*02:02 | 0.58 |
| DRB1*14:03:01-DQB1*03:01:01-DPB1*05:01:01 | 0.58 |
| DRB1*04:01:01-DQB1*03:01:01-DPB1*02:01:02 | 0.58 |
| DRB1*13:01:01-DQB1*06:03:01-DPB1*02:01:02 | 0.58 |
| DRB1*04:01:01-DQB1*03:01:01-DPB1*04:01:01 | 0.58 |
| DRB1*14:06:01-DQB1*03:01:01-DPB1*13:01:01 | 0.58 |
| DRB1*07:01:01-DQB1*02:02:01-DPB1*04:02:01 | 0.58 |
| DRB1*13:02:01-DQB1*06:09:01-DPB1*03:01:01 | 0.58 |
| DRB1*04:06:01-DQB1*03:02:01-DPB1*03:01:01 | 0.58 |
| DRB1*07:01:01-DQB1*02:02:01-DPB1*02:01:02 | 0.58 |

HF, haplotype frequency
