# Supplementary material for: Allele and haplotype frequencies of human leukocyte antigen-A, -B, -C, -DRB1, -DRB3/4/5, -DQA1, -DQB1, -DPA1, and -DPB1 by next generation sequencing-based typing in Koreans in South Korea
Source: PLoS One. 2021 Jun 21;16(6):e0253619. doi: 10.1371/journal.pone.0253619 (PMC8216545; doi:10.1371/journal.pone.0253619)
Supplement: S6 Table — (DOCX) [file pone.0253619.s006.docx]

**S6 Table.** The entire configuration file (.ini)

;; comment out or change as desired

;; 1 = true, 0 = false

[General]

debug=0

[ParseGenotypeFile]

untypedAllele=****

;; designates field name that holds allele data

alleleDesignator=*

;; valid fields for sample data block

validSampleFields=*a_1

*a_2

*c_1

*c_2

*b_1

*b_2

*drb1_1

*drb1_2

*dqa1_1

*dqa1_2

*dqb1_1

*dqb1_2

*dpa1_1

*dpa1_2

*dpb1_1

*dpb1_2

[HardyWeinberg]

lumpBelow=5

[HardyVeinbergGuoThompson]

dememorizationSteps=2000

samplingNum=1000

samplingSize=1000

[HomozygosityEWSlatkinExact]

;; use section if the Monte Carlo approximation to the Slatkin exact

;; Ewens-Watterson test should be run

numReplicates=10000

[Emhaplofreq]

;; comma (',') separated haplotypes blocks for which to estimate

;; haplotypes, within each "block", each locus is separated by colons

;; (':') e.g. dqa1:dpb1,drb1:dqb1, means to est. of haplotypes for

;; 'dqa1' and 'dpb1' loci followed by est. of haplotypes for 'drb1'

;; and 'dqb1' loci. A wildcard entry '*' means estimate haplotypes

;; for the entire loci as specified in the original file column order

;;lociToEstHaplo=a:b:drb1,a:b:c,drb1:dqa1:dpb1,drb1:dqb1:dpb1

lociToEstHaplo=a:b:drb1,a:b:c,a:b:c:drb1,a:b:c:drb1:dqb1,drb1:dqb1:dpb1,drb1:dqa1:dqb1:dpa1:dpb1,a:b:c:drb1:dqa1:dqb1,a:b:c:drb1:dqb1:dpb1

;; analogous to `lociToEst' except for linkage disequilibrium (LD)

;;lociToEstLD=

;; pairwise estimates can be run in two ways: with or without

;; permutation test.

;; if LD *and* permutation test for all pairwise loci are desired,

;; then set both options '1' (true); the separate

;; 'allPairwiseLDWithHaplo' and 'allPairwiseLDWithHaploWithPermu'

;; options are now obsolete

;; estimate LD for all pairwise loci?

allPairwiseLD=1

;; with permutation test?

allPairwiseLDWithPermu=0
