# Supplementary material for: Allele and haplotype frequencies of human leukocyte antigen-A, -B, -C, -DRB1, -DRB3/4/5, -DQA1, -DQB1, -DPA1, and -DPB1 by next generation sequencing-based typing in Koreans in South Korea
Source: PLoS One. 2021 Jun 21;16(6):e0253619. doi: 10.1371/journal.pone.0253619 (PMC8216545; doi:10.1371/journal.pone.0253619)
Supplement: S7 Table — (DOCX) [file pone.0253619.s007.docx]

**S7 Table.** Alleles resolved on ambiguous allele combinations of the IPD-IMGT/HLA database (Release version 3.42.0) (n = 173)

| **IPD-IMGT/HLA ambiguous allele combinations** | **The number of allele in each G group** | **2n** | **%** |  | **TruSight HLA typing v2** | **2n** | **%** |
| --- | --- | --- | --- | --- | --- | --- | --- |
| **HLA-C (exon 2+3)** |  |  |  |  | **HLA-C** |  |  |
| C*07:01:02/*07:06:01 | 187 | 9 | 2.60 |  | C*07:01:02 | 1 | 0.29 |
|  |  |  |  |  | C*07:06:01 | 8 | 2.31 |
| C*08:01:01/*08:22:01 | 42 | 15 | 4.40 |  | C*08:01:01 | 12 | 3.47 |
|  |  |  |  |  | C*08:22:01 | 3 | 0.87 |
|  |  |  |  |  |  |  |  |
| **LA-DRB1 (exon 2)** |  |  |  |  | **HLA-DRB1** |  |  |
| DRB1*14:01:01/*14:54:01 | 17 | 12 | 3.47 |  | DRB1*14:54:01 | 12 | 3.47 |
|  |  |  |  |  |  |  |  |
| **HLA-DQA1 (exon 2)** |  |  |  |  | **HLA-DQA1** |  |  |
| DQA1*01:01:01/*01:04:01/01:05:01 | 24 | 52 | 15.03 |  | DQA1*01:01:01 | 23 | 6.65 |
|  |  |  |  |  | DQA1*01:04:01 | 27 | 7.80 |
|  |  |  |  |  | DQA1*01:05:01 | 2 | 0.58 |
| DQA1*03:01:01/*03:02:01/*03:03:01 | 25 | 96 | 27.75 |  | DQA1*03:01:01 | 39 | 11.27 |
|  |  |  |  |  | DQA1*03:02:01 | 19 | 5.49 |
|  |  |  |  |  | DQA1*03:03:01 | 37 | 10.69 |
| DQA1*05:01:01/*05:03:01/*05:05:01/*05:06:01/*05:07/*05:08 | 51 | 45 | 13.01 |  | DQA1*05:01:01 | 11 | 3.18 |
|  |  |  |  |  | DQA1*05:03:01 | 5 | 1.45 |
|  |  |  |  |  | DQA1*05:05:01 | 16 | 4.62 |
|  |  |  |  |  | DQA1*05:06:01 | 4 | 1.16 |
|  |  |  |  |  | DQA1*05:07 | 2 | 0.58 |
|  |  |  |  |  | DQA1*05:08 | 7 | 2.02 |
|  |  |  |  |  |  |  |  |
| **HLA-DQB1 (exon 2)** |  |  |  |  | **HLA-DQB1** |  |  |
| DQB1*02:01:01/*02:02:01 | 81 | 34 | 9.83 |  | DQB1*02:01:01 | 8 | 2.31 |
|  |  |  |  |  | DQB1*02:02:01 | 26 | 7.51 |
|  |  |  |  |  |  |  |  |
| **HLA-DPB1 (exon 2)** |  |  |  |  | **HLA-DPB1** |  |  |
| DPB1*02:01:02/*414:01 | 96 | 87 | 25.43 |  | DPB1*02:01:02 | 87 | 25.14 |
|  |  |  |  |  | DPB1*414:01 | 1 | 0.29 |
| DPB1*03:01:01/*104:01 | 48 | 17 | 4.91 |  | DPB1*03:01:01 | 16 | 4.62 |
|  |  |  |  |  | DPB1*104:01 | 1 | 0.29 |
| DPB1*05:01:01/*135:01 | 36 | 118 | 34.39 |  | DPB1*05:01:01 | 118 | 34.10 |
|  |  |  |  |  | DPB1*135:01 | 1 | 0.29 |
