# Supplementary material for: Allele and haplotype frequencies of human leukocyte antigen-A, -B, -C, -DRB1, -DRB3/4/5, -DQA1, -DQB1, -DPA1, and -DPB1 by next generation sequencing-based typing in Koreans in South Korea
Source: PLoS One. 2021 Jun 21;16(6):e0253619. doi: 10.1371/journal.pone.0253619 (PMC8216545; doi:10.1371/journal.pone.0253619)
Supplement: S8 Table — (DOCX) [file pone.0253619.s008.docx]

**S8 Table.** The Hardy-Weinberg equilibrium of HLA-A, -B, -C, -DRB1, -DQA1, -DQB1, -DPA1, and -DPB1 loci in South Korea population

| Locus | All homozygotes | | | All heterozygotes | | |
| --- | --- | --- | --- | --- | --- | --- |
|  | Observed | Expected | p-value | Observed | Expected | p-value |
| A | 25 | 22.87 | 0.6565 | 148 | 150.13 | 0.8622 |
| B | 4 | 8.42 | 0.1278 | 169 | 164.58 | 0.7305 |
| C | 17 | 17.14 | 0.9738 | 156 | 155.86 | 0.9913 |
| DRB1 | 11 | 10.49 | 0.8752 | 162 | 162.51 | 0.9682 |
| DQA1 | 21 | 16.58 | 0.2778 | 152 | 156.42 | 0.7238 |
| DQB1 | 17 | 16.13 | 0.8291 | 156 | 156.87 | 0.9448 |
| DPA1 | 67 | 65.52 | 0.8552 | 106 | 107.48 | 0.8867 |
| DPB1 | 35 | 34.86 | 0.9816 | 138 | 138.14 | 0.9908 |
