# Supplementary material for: Allele and haplotype frequencies of human leukocyte antigen-A, -B, -C, -DRB1, -DRB3/4/5, -DQA1, -DQB1, -DPA1, and -DPB1 by next generation sequencing-based typing in Koreans in South Korea
Source: PLoS One. 2021 Jun 21;16(6):e0253619. doi: 10.1371/journal.pone.0253619 (PMC8216545; doi:10.1371/journal.pone.0253619)
Supplement: S9 Table — (DOCX) [file pone.0253619.s009.docx]

**S9 Table**. Allele frequencies of HLA-A, -B, and -C in South Koreans (N=173)

| **HLA alleles** | **2n** | **%** |  | **HLA alleles** | **2n** | **%** |  | **HLA alleles** | **2n** | **%** |
| --- | --- | --- | --- | --- | --- | --- | --- | --- | --- | --- |
| A*01:01:01 | 3 | 0.87 |  | B*15:17:01 | 1 | 0.29 |  | B*67:01:02 | 1 | 0.29 |
| A*02:01:01 | 79 | 22.83 |  | B*15:18:01 | 4 | 1.16 |  |  |  |  |
| A*02:03:01 | 3 | 0.87 |  | B*15:27:01 | 2 | 0.58 |  | C*01:02:01 | 72 | 20.81 |
| A*02:06:01 | 22 | 6.36 |  | B*27:04:01 | 1 | 0.29 |  | C*01:03:01 | 3 | 0.87 |
| A*02:07:01 | 18 | 5.20 |  | B*27:05:02 | 11 | 3.18 |  | C*02:02:02 | 4 | 1.16 |
| A*02:10:01 | 1 | 0.29 |  | B*35:01:01 | 18 | 5.20 |  | C*03:02:01 | 2 | 0.58 |
| A*03:01:01 | 4 | 1.16 |  | B*35:03:01 | 1 | 0.29 |  | C*03:02:02 | 19 | 5.49 |
| A*03:02:01 | 3 | 0.87 |  | B*37:01:01 | 2 | 0.58 |  | C*03:03:01 | 46 | 13.29 |
| A*11:01:01 | 45 | 13.01 |  | B*38:02:01 | 5 | 1.45 |  | C*03:04:01 | 32 | 9.25 |
| A*24:02:01 | 55 | 15.90 |  | B*39:01:01 | 3 | 0.87 |  | C*04:01:01 | 21 | 6.07 |
| A*24:20:01 | 1 | 0.29 |  | B*40:01:02 | 11 | 3.18 |  | C*05:01:01 | 4 | 1.16 |
| A*26:01:01 | 14 | 4.05 |  | B*40:02:01 | 15 | 4.34 |  | C*06:02:01 | 17 | 4.91 |
| A*26:02:01 | 8 | 2.31 |  | B*40:03:01 | 3 | 0.87 |  | C*07:01:02 | 1 | 0.29 |
| A*29:01:01 | 3 | 0.87 |  | B*40:06:01 | 7 | 2.02 |  | C*07:02:01 | 39 | 11.27 |
| A*30:01:01 | 10 | 2.89 |  | B*44:02:01 | 4 | 1.16 |  | C*07:04:01 | 4 | 1.16 |
| A*30:04:01 | 5 | 1.45 |  | B*44:03:01 | 20 | 5.78 |  | C*07:06:01 | 8 | 2.31 |
| A*31:01:02 | 12 | 3.47 |  | B*44:03:02 | 9 | 2.60 |  | C*08:01:01 | 12 | 3.47 |
| A*32:01:01 | 3 | 0.87 |  | B*46:01:01 | 21 | 6.07 |  | C*08:02:01 | 2 | 0.58 |
| A*33:03:01 | 56 | 16.18 |  | B*48:01:01 | 13 | 3.76 |  | C*08:03:01 | 6 | 1.73 |
| A*68:01:02 | 1 | 0.29 |  | B*51:01:01 | 17 | 4.91 |  | C*08:06 | 1 | 0.29 |
|  |  |  |  | B*51:02:01 | 4 | 1.16 |  | C*08:22:01 | 3 | 0.87 |
| B*07:02:01 | 17 | 4.91 |  | B*52:01:01 | 5 | 1.45 |  | C*12:02:02 | 5 | 1.45 |
| B*07:05:01 | 3 | 0.87 |  | B*54:01:01 | 27 | 7.80 |  | C*12:03:01 | 2 | 0.58 |
| B*08:01:01 | 3 | 0.87 |  | B*54:19 | 1 | 0.29 |  | C*14:02:01 | 11 | 3.18 |
| B*13:01:01 | 11 | 3.18 |  | B*55:02:01 | 6 | 1.73 |  | C*14:03:01 | 19 | 5.49 |
| B*13:02:01 | 17 | 4.91 |  | B*55:07 | 1 | 0.29 |  | C*14:39 | 1 | 0.29 |
| B*14:01:01 | 2 | 0.58 |  | B*56:01:01 | 2 | 0.58 |  | C*15:02:01 | 9 | 2.60 |
| B*15:01:01 | 38 | 10.98 |  | B*58:01:01 | 20 | 5.78 |  | C*15:05:02 | 3 | 0.87 |
| B*15:07:01 | 4 | 1.16 |  | B*59:01:01 | 10 | 2.89 |  |  |  |  |
| B*15:11:01 | 5 | 1.45 |  | B*67:01:01 | 1 | 0.29 |  |  |  |  |
