# Supplementary material for: Allele and haplotype frequencies of human leukocyte antigen-A, -B, -C, -DRB1, -DRB3/4/5, -DQA1, -DQB1, -DPA1, and -DPB1 by next generation sequencing-based typing in Koreans in South Korea
Source: PLoS One. 2021 Jun 21;16(6):e0253619. doi: 10.1371/journal.pone.0253619 (PMC8216545; doi:10.1371/journal.pone.0253619)
Supplement: S10 Table — (DOCX) [file pone.0253619.s010.docx]

**S10 Table**. Allele frequencies of HLA-DRB1, -DQA1, -DQB1, -DPA1, and -DPB1 in South Koreans (N=173)

| **HLA alleles** | **2n** | **%** |  | **HLA alleles** | **2n** | **%** |  | **HLA alleles** | **2n** | **%** |
| --- | --- | --- | --- | --- | --- | --- | --- | --- | --- | --- |
| DRB1*01:01:01 | 20 | 5.78 |  | DRB1*16:02:01 | 1 | 0.29 |  | DQB1*05:01:01 | 23 | 6.65 |
| DRB1*03:01:01 | 8 | 2.31 |  |  |  |  |  | DQB1*05:02:01 | 12 | 3.47 |
| DRB1*04:01:01 | 4 | 1.16 |  | DQA1*01:01:01 | 23 | 6.65 |  | DQB1*05:03:01 | 18 | 5.20 |
| DRB1*04:03:01 | 12 | 3.47 |  | DQA1*01:02:01 | 55 | 15.90 |  | DQB1*06:01:01 | 46 | 13.29 |
| DRB1*04:04:01 | 2 | 0.58 |  | DQA1*01:02:02 | 1 | 0.29 |  | DQB1*06:02:01 | 22 | 6.36 |
| DRB1*04:05:01 | 29 | 8.38 |  | DQA1*01:03:01 | 49 | 14.16 |  | DQB1*06:03:01 | 4 | 1.16 |
| DRB1*04:06:01 | 18 | 5.20 |  | DQA1*01:04:01 | 27 | 7.80 |  | DQB1*06:04:01 | 21 | 6.07 |
| DRB1*04:07:01 | 1 | 0.29 |  | DQA1*01:05:01 | 2 | 0.58 |  | DQB1*06:09:01 | 11 | 3.18 |
| DRB1*04:10:01 | 3 | 0.87 |  | DQA1*02:01:01 | 27 | 7.80 |  |  |  |  |
| DRB1*07:01:01 | 27 | 7.80 |  | DQA1*03:01:01 | 39 | 11.27 |  | DPA1*01:03:01 | 152 | 43.93 |
| DRB1*08:02:01 | 9 | 2.60 |  | DQA1*03:02:01 | 19 | 5.49 |  | DPA1*01:04 | 1 | 0.29 |
| DRB1*08:03:02 | 40 | 11.56 |  | DQA1*03:03:01 | 37 | 10.69 |  | DPA1*02:01:01 | 54 | 15.61 |
| DRB1*09:01:02 | 19 | 5.49 |  | DQA1*03:03:02 | 1 | 0.29 |  | DPA1*02:02:02 | 139 | 40.17 |
| DRB1*10:01:01 | 2 | 0.58 |  | DQA1*04:01:01 | 3 | 0.87 |  |  |  |  |
| DRB1*11:01:01 | 14 | 4.05 |  | DQA1*05:01:01 | 11 | 3.18 |  | DPB1*02:01:02 | 87 | 25.14 |
| DRB1*11:45 | 1 | 0.29 |  | DQA1*05:03:01 | 5 | 1.45 |  | DPB1*02:02:01 | 18 | 5.20 |
| DRB1*12:01:01 | 17 | 4.91 |  | DQA1*05:05:01 | 16 | 4.62 |  | DPB1*03:01:01 | 16 | 4.62 |
| DRB1*12:02:01 | 12 | 3.47 |  | DQA1*05:06:01 | 4 | 1.16 |  | DPB1*04:01:01 | 21 | 6.07 |
| DRB1*13:01:01 | 4 | 1.16 |  | DQA1*05:07 | 2 | 0.58 |  | DPB1*04:02:01 | 30 | 8.67 |
| DRB1*13:02:01 | 32 | 9.25 |  | DQA1*05:08 | 7 | 2.02 |  | DPB1*05:01:01 | 118 | 34.10 |
| DRB1*13:198 | 1 | 0.29 |  | DQA1*06:01:01 | 18 | 5.20 |  | DPB1*09:01:01 | 11 | 3.18 |
| DRB1*14:03:01 | 3 | 0.87 |  |  |  |  |  | DPB1*13:01:01 | 20 | 5.78 |
| DRB1*14:04:01 | 1 | 0.29 |  | DQB1*02:01:01 | 8 | 2.31 |  | DPB1*14:01:01 | 8 | 2.31 |
| DRB1*14:05:01 | 13 | 3.76 |  | DQB1*02:02:01 | 26 | 7.51 |  | DPB1*15:01:01 | 1 | 0.29 |
| DRB1*14:06:01 | 3 | 0.87 |  | DQB1*03:01:01 | 57 | 16.47 |  | DPB1*17:01:01 | 11 | 3.18 |
| DRB1*14:07:01 | 2 | 0.58 |  | DQB1*03:02:01 | 41 | 11.85 |  | DPB1*36:01 | 1 | 0.29 |
| DRB1*14:54:01 | 12 | 3.47 |  | DQB1*03:03:02 | 21 | 6.07 |  | DPB1*38:01:01 | 1 | 0.29 |
| DRB1*14:142 | 1 | 0.29 |  | DQB1*03:13 | 1 | 0.29 |  | DPB1*104:01 | 1 | 0.29 |
| DRB1*15:01:01 | 23 | 6.65 |  | DQB1*04:01:01 | 29 | 8.38 |  | DPB1*135:01 | 1 | 0.29 |
| DRB1*15:02:01 | 12 | 3.47 |  | DQB1*04:02:01 | 6 | 1.73 |  | DPB1*414:01 | 1 | 0.29 |
