# Supplementary material for: Allele and haplotype frequencies of human leukocyte antigen-A, -B, -C, -DRB1, -DRB3/4/5, -DQA1, -DQB1, -DPA1, and -DPB1 by next generation sequencing-based typing in Koreans in South Korea
Source: PLoS One. 2021 Jun 21;16(6):e0253619. doi: 10.1371/journal.pone.0253619 (PMC8216545; doi:10.1371/journal.pone.0253619)
Supplement: S11 Table — (DOCX) [file pone.0253619.s011.docx]

**S11 Table.** Haplotype frequencies of HLA-A, -B, and -C except for overlapping parts of Table 2 (>1%)

| HLA haplotypes | HF (%) |
| --- | --- |
| A*11:01:01-B*54:01:01-C*01:02:01 | 2.49 |
| A*24:02:01-B*59:01:01-C*01:02:01 | 2.31 |
| A*02:01:01-B*40:02:01-C*03:04:01 | 2.02 |
| A*02:01:01-B*13:01:01-C*03:04:01 | 1.45 |
| A*02:01:01-B*15:11:01-C*03:03:01 | 1.45 |
| A*02:01:01-B*27:05:02-C*01:02:01 | 1.44 |
| A*11:01:01-B*35:01:01-C*03:03:01 | 1.13 |
| A*02:06:01-B*46:01:01-C*01:02:01 | 1.12 |

HF, haplotype frequency
