# Supplementary material for: Allele and haplotype frequencies of human leukocyte antigen-A, -B, -C, -DRB1, -DRB3/4/5, -DQA1, -DQB1, -DPA1, and -DPB1 by next generation sequencing-based typing in Koreans in South Korea
Source: PLoS One. 2021 Jun 21;16(6):e0253619. doi: 10.1371/journal.pone.0253619 (PMC8216545; doi:10.1371/journal.pone.0253619)
Supplement: S12 Table — (DOCX) [file pone.0253619.s012.docx]

**S12 Table**. Haplotype frequencies of HLA-DRB1, -DQA1, -DQB1, -DPA1, and -DPB1 (>1%)

| HLA haplotypes | HF (%) |
| --- | --- |
| DRB1*08:03:02-DQA1*01:03:01-DQB1*06:01:01-DPA1*02:02:02-DPB1*05:01:01 | 5.41 |
| DRB1*04:05:01-DQA1*03:03:01-DQB1*04:01:01-DPA1*02:02:02-DPB1*05:01:01 | 4.34 |
| DRB1*13:02:01-DQA1*01:02:01-DQB1*06:04:01-DPA1*01:03:01-DPB1*04:01:01 | 4.05 |
| DRB1*09:01:02-DQA1*03:02-DQB1*03:03:02-DPA1*02:02:02-DPB1*05:01:01 | 3.06 |
| DRB1*15:01:01-DQA1*01:02:01-DQB1*06:02:01-DPA1*01:03:01-DPB1*02:01:02 | 3.04 |
| DRB1*07:01:01-DQA1*02:01-DQB1*02:02:01-DPA1*02:01:01-DPB1*17:01:01 | 2.89 |
| DRB1*04:06:01-DQA1*03:01:01-DQB1*03:02:01-DPA1*02:02:02-DPB1*05:01:01 | 2.85 |
| DRB1*12:02:01-DQA1*06:01:01-DQB1*03:01:01-DPA1*02:02:02-DPB1*05:01:01 | 2.60 |
| DRB1*15:02:01-DQA1*01:03:01-DQB1*06:01:01-DPA1*02:01:01-DPB1*09:01:01 | 2.60 |
| DRB1*01:01:01-DQA1*01:01:01-DQB1*05:01:01-DPA1*01:03:01-DPB1*04:02:01 | 2.48 |
| DRB1*01:01:01-DQA1*01:01:01-DQB1*05:01:01-DPA1*02:02:02-DPB1*05:01:01 | 2.40 |
| DRB1*15:01:01-DQA1*01:02:01-DQB1*06:02:01-DPA1*02:02:02-DPB1*05:01:01 | 2.22 |
| DRB1*13:02:01-DQA1*01:02:01-DQB1*06:09:01-DPA1*01:03:01-DPB1*02:01:02 | 1.73 |
| DRB1*04:03:01-DQA1*03:01:01-DQB1*03:02:01-DPA1*01:03:01-DPB1*02:01:02 | 1.73 |
| DRB1*04:05:01-DQA1*03:03:01-DQB1*04:01:01-DPA1*01:03:01-DPB1*04:02:01 | 1.48 |
| DRB1*07:01:01-DQA1*02:01-DQB1*02:02:01-DPA1*02:02:02-DPB1*05:01:01 | 1.45 |
| DRB1*07:01:01-DQA1*02:01-DQB1*02:02:01-DPA1*02:01:01-DPB1*13:01:01 | 1.45 |
| DRB1*11:01:01-DQA1*05:05:01-DQB1*03:01:01-DPA1*01:03:01-DPB1*02:01:02 | 1.45 |
| DRB1*08:02:01-DQA1*03:01:01-DQB1*03:02:01-DPA1*02:02:02-DPB1*05:01:01 | 1.45 |
| DRB1*04:06:01-DQA1*03:01:01-DQB1*03:02:01-DPA1*01:03:01-DPB1*02:01:02 | 1.45 |
| DRB1*08:03:02-DQA1*01:03:01-DQB1*06:01:01-DPA1*02:02:02-DPB1*02:02 | 1.43 |
| DRB1*04:05:01-DQA1*03:03:01-DQB1*04:01:01-DPA1*01:03:01-DPB1*02:01:02 | 1.40 |
| DRB1*03:01:01-DQA1*05:01:01-DQB1*02:01:01-DPA1*01:03:01-DPB1*04:01:01 | 1.16 |
| DRB1*08:03:02-DQA1*06:01:01-DQB1*03:01:01-DPA1*01:03:01-DPB1*02:01:02 | 1.16 |

HF, haplotype frequency
