# Supplementary material for: Allele and haplotype frequencies of human leukocyte antigen-A, -B, -C, -DRB1, -DRB3/4/5, -DQA1, -DQB1, -DPA1, and -DPB1 by next generation sequencing-based typing in Koreans in South Korea
Source: PLoS One. 2021 Jun 21;16(6):e0253619. doi: 10.1371/journal.pone.0253619 (PMC8216545; doi:10.1371/journal.pone.0253619)
Supplement: S13 Table — (DOCX) [file pone.0253619.s013.docx]

**S13 Table.** Haplotype frequencies of 2-locus haplotypes of HLA class II genes (>1%)

| DRB1-DRB3/4/5 | HF (%) |  | DQA1-DQB1 | HF (%) |  | DPA1-DPB1 | HF (%) |
| --- | --- | --- | --- | --- | --- | --- | --- |
| DRB1*04:05:01-DRB4*01:03:01 | 12.72 |  | DQA1*01:03:01-DQB1*06:01:01 | 13.01 |  | DPA1*02:02:02-DPB1*05:01:01 | 30.83 |
| DRB1*13:02:01-DRB3*03:01:01 | 9.25 |  | DQA1*03:01:01-DQB1*03:02:01 | 11.27 |  | DPA1*01:03:01-DPB1*02:01:02 | 22.01 |
| DRB1*04:06:01-DRB4*01:03:01 | 8.09 |  | DQA1*03:03:01-DQB1*04:01:01 | 8.09 |  | DPA1*01:03:01-DPB1*04:02:01 | 8.67 |
| DRB1*07:01:01-DRB4*01:03:01 | 6.36 |  | DQA1*02:01-DQB1*02:02:01 | 7.51 |  | DPA1*01:03:01-DPB1*04:01:01 | 6.07 |
| DRB1*15:01:01-DRB5*01:01:01 | 6.36 |  | DQA1*01:02:01-DQB1*06:02:01 | 6.36 |  | DPA1*02:01:01-DPB1*13:01:01 | 4.59 |
| DRB1*09:01:02-DRB4*01:03:02 | 5.78 |  | DQA1*01:01:01-DQB1*05:01:01 | 6.07 |  | DPA1*01:03:01-DPB1*03:01:01 | 3.32 |
| DRB1*15:02:01-DRB5*01:02 | 5.78 |  | DQA1*01:02:01-DQB1*06:04:01 | 6.07 |  | DPA1*02:01:01-DPB1*09:01:01 | 3.18 |
| DRB1*12:01:01-DRB3*02:02:01 | 5.20 |  | DQA1*03:02-DQB1*03:03:02 | 5.49 |  | DPA1*02:02:02-DPB1*02:01:02 | 3.14 |
| DRB1*14:54:01-DRB3*02:02:01 | 5.20 |  | DQA1*06:01:01-DQB1*03:01:01 | 5.20 |  | DPA1*02:02:02-DPB1*02:02 | 3.13 |
| DRB1*12:02:01-DRB3*03:01:03 | 4.62 |  | DQA1*01:04:01-DQB1*05:03:01 | 4.62 |  | DPA1*02:01:01-DPB1*17:01:01 | 2.89 |
| DRB1*04:03:01-DRB4*01:03:01 | 3.47 |  | DQA1*05:05:01-DQB1*03:01:01 | 4.05 |  | DPA1*02:01:01-DPB1*05:01:01 | 2.64 |
| DRB1*11:01:01-DRB3*02:02:01 | 3.47 |  | DQA1*01:02:01-DQB1*06:09:01 | 3.18 |  | DPA1*02:01:01-DPB1*14:01:01 | 2.31 |
| DRB1*03:01:01-DRB3*02:02:01 | 2.89 |  | DQA1*01:04:01-DQB1*05:02:01 | 3.18 |  | DPA1*01:03:01-DPB1*02:02 | 2.07 |
| DRB1*09:01:02-DRB4*01:03:01 | 2.89 |  | DQA1*05:01:01-DQB1*02:01:01 | 2.31 |  | DPA1*02:02:02-DPB1*03:01:01 | 1.31 |
| DRB1*14:05:01-DRB3*02:02:01 | 2.89 |  | DQA1*05:08-DQB1*03:01:01 | 2.02 |  | DPA1*02:02:02-DPB1*13:01:01 | 1.19 |
| DRB1*11:01:01-DRB3*01:01:02 | 1.73 |  | DQA1*05:03-DQB1*03:01:01 | 1.45 |  |  |  |
| DRB1*13:01:01-DRB3*01:01:02 | 1.73 |  | DQA1*03:03:01-DQB1*03:01:01 | 1.16 |  |  |  |
| DRB1*14:03:01-DRB3*01:01:02 | 1.73 |  | DQA1*01:03:01-DQB1*06:03:01 | 1.16 |  |  |  |
| DRB1*14:07:01-DRB3*02:02:01 | 1.73 |  | DQA1*03:03:01-DQB1*04:02:01 | 1.16 |  |  |  |
|  |  |  | DQA1*05:06-DQB1*03:01:01 | 1.16 |  |  |  |

In DRB1/3/4/5 locus, not all samples are representative for secondary DR loci. Only samples containing one copy of the secondary locus could been evaluated. HF, haplotype frequency
