# Supplementary material for: Allele and haplotype frequencies of human leukocyte antigen-A, -B, -C, -DRB1, -DRB3/4/5, -DQA1, -DQB1, -DPA1, and -DPB1 by next generation sequencing-based typing in Koreans in South Korea
Source: PLoS One. 2021 Jun 21;16(6):e0253619. doi: 10.1371/journal.pone.0253619 (PMC8216545; doi:10.1371/journal.pone.0253619)
Supplement: S14 Table — (DOCX) [file pone.0253619.s014.docx]

**S14 Table.** Expected PCR-SBT ambiguities (described in the IPD-IMGT/HLA database) solved by the NGS assay (n = 173)

| **Groups** |  | **Allele 1** | **%** |  | **Allele 2** | **%** |  | **Allele 3** | **%** |  | **Allele 4** | **%** |  | **Allele 5** | **%** |  | **Allele 6** | **%** |
| --- | --- | --- | --- | --- | --- | --- | --- | --- | --- | --- | --- | --- | --- | --- | --- | --- | --- | --- |
|  |  | **HLA-C** |  |  |  |  |  |  |  |  |  |  |  |  |  |  |  |  |
| 1 |  | C*07:01:02 | 0.29 |  | C*07:06:01 | 2.31 |  |  |  |  |  |  |  |  |  |  |  |  |
| 2 |  | C*08:01:01 | 3.47 |  | C*08:22:01 | 0.87 |  |  |  |  |  |  |  |  |  |  |  |  |
|  |  |  |  |  |  |  |  |  |  |  |  |  |  |  |  |  |  |  |
|  |  | **HLA-DRB1** |  |  |  |  |  |  |  |  |  |  |  |  |  |  |  |  |
| 3 |  | DRB1*14:01:01 | - |  | DRB1*14:54:01 | 3.47 |  |  |  |  |  |  |  |  |  |  |  |  |
|  |  |  |  |  |  |  |  |  |  |  |  |  |  |  |  |  |  |  |
|  |  | **HLA-DQA1** |  |  |  |  |  |  |  |  |  |  |  |  |  |  |  |  |
| 4 |  | DQA1*01:01:01 | 6.65 |  | DQA1*01:04:01 | 7.80 |  | DQA1*01:05:01 | 0.58 |  |  |  |  |  |  |  |  |  |
| 5 |  | DQA1*03:01:01 | 11.27 |  | DQA1*03:02:01 | 5.49 |  | DQA1*03:03:01 | 10.69 |  |  |  |  |  |  |  |  |  |
| 6 |  | DQA1*05:01:01 | 3.18 |  | DQA1*05:03:01 | 1.45 |  | DQA1*05:05:01 | 4.62 |  | DQA1*05:06:01 | 1.16 |  | DQA1*05:07 | 0.58 |  | DQA1*05:08 | 2.02 |
|  |  |  |  |  |  |  |  |  |  |  |  |  |  |  |  |  |  |  |
|  |  | **HLA-DQB1** |  |  |  |  |  |  |  |  |  |  |  |  |  |  |  |  |
| 7 |  | DQB1*02:01:01 | 2.31 |  | DQB1*02:02:01 | 7.51 |  |  |  |  |  |  |  |  |  |  |  |  |
|  |  |  |  |  |  |  |  |  |  |  |  |  |  |  |  |  |  |  |
|  |  | **HLA-DPB1** |  |  |  |  |  |  |  |  |  |  |  |  |  |  |  |  |
| 8 |  | DPB1*02:01:02 | 25.14 |  | DPB1*414:01 | 0.29 |  |  |  |  |  |  |  |  |  |  |  |  |
| 9 |  | DPB1*03:01:01 | 4.62 |  | DPB1*104:01 | 0.29 |  |  |  |  |  |  |  |  |  |  |  |  |
| 10 |  | DPB1*05:01:01 | 34.10 |  | DPB1*135:01 | 0.29 |  |  |  |  |  |  |  |  |  |  |  |  |
