# Supplementary material for: Allele and haplotype frequencies of human leukocyte antigen-A, -B, -C, -DRB1, -DRB3/4/5, -DQA1, -DQB1, -DPA1, and -DPB1 by next generation sequencing-based typing in Koreans in South Korea
Source: PLoS One. 2021 Jun 21;16(6):e0253619. doi: 10.1371/journal.pone.0253619 (PMC8216545; doi:10.1371/journal.pone.0253619)
Supplement: S15 Table — (DOCX) [file pone.0253619.s015.docx]

**S15 Table.** HLA-A allele frequencies of 16 populations*

| **alleles** | **South Korean** | **Japanese**** | **Han Chinese** | **Southeast Asian** | **Southwest Asian** | **Oceanian** | **Australian** | **Northern Sami** | **Southern Sami** | **Non-Sami Swedish** | **Finnish** | **European** | **South American** | **North American** | **North African** | **Sub-Saharan African** |
| --- | --- | --- | --- | --- | --- | --- | --- | --- | --- | --- | --- | --- | --- | --- | --- | --- |
| **A*0101** | **0.9** | 0.4 | 6.0 | 1.0 | 9.0 |  | 2.0 | 8.3 | 9.3 | 11.5 | 8.9 | 16.0 |  | 4.0 | 14.0 | 6.0 |
| **A*0201** | **22.8** | 11.6 | 15.0 | 7.0 | 16.0 | 14.0 | 13.0 | 23.6 | 26.0 | 37.5 | 34.4 | 27.0 | 22.0 | 14.0 | 18.0 | 10.0 |
| **A*0203** | **0.9** | 0.1 |  |  |  |  |  |  |  |  |  |  |  |  |  |  |
| **A*0206** | **6.4** | 9.1 | 7.0 | 5.0 |  | 5.0 |  | 1.4 | 1.2 |  |  |  |  | 11.0 |  |  |
| **A*0207** | **5.2** | 3.5 |  |  |  |  |  |  |  |  |  |  |  |  |  |  |
| **A*0210** | **0.3** | 0.4 |  |  |  |  |  |  |  |  |  |  |  |  |  |  |
| **A*0301** | **1.2** | 0.4 | 4.0 | 1.0 | 5.0 | 1.0 | 1.0 | 31.3 | 24.8 | 16.1 | 25.0 | 14.0 |  | 4.0 | 4.0 | 5.0 |
| **A*0302** | **0.9** | 0.1 |  |  |  |  |  |  |  |  |  |  |  |  |  |  |
| **A*1101** | **13.0** | 9.1 | 9.0 | 21.0 | 10.0 | 19.0 | 13.0 | 1.0 | 5.8 | 4.8 | 5.0 | 7.0 |  | 2.0 | 4.0 | 1.0 |
| **A*2402** | **15.9** | 36.5 | 22.0 | 30.0 | 8.0 | 27.0 | 26.0 | 21.2 | 13.2 | 7.5 | 9.4 | 8.0 | 13.0 | 38.0 | 5.0 | 2.0 |
| **A*2420** | **0.3** | 0.7 |  |  |  |  |  |  |  |  |  |  |  |  |  |  |
| **A*2601** | **4.1** | 7.4 | 4.0 | 4.0 | 8.0 | 3.0 |  | 3.1 | 3.1 | 2.6 | 1.1 | 3.0 |  | 1.0 | 2.0 | 1.0 |
| **A*2602** | **2.3** | 1.8 |  |  |  |  |  |  |  |  |  |  |  |  |  |  |
| **A*2901** | **0.9** | 0.0 |  | 1.0 | 3.0 |  |  |  | 0.7 |  | 1.1 |  |  |  |  | 1.0 |
| **A*3001** | **2.9** | 0.2 |  |  |  |  |  |  |  |  | 0.6 |  |  |  |  |  |
| **A*3004** | **1.5** | 0.0 |  |  |  |  |  |  |  |  |  |  |  |  |  |  |
| **A*3101** | **3.5** | 8.4 | 7.0 | 2.0 | 2.0 |  | 2.0 | 5.9 | 5.8 | 2.6 | 5.0 | 3.0 | 36.0 | 5.0 | 2.0 | 1.0 |
| **A*3201** | **0.9** | 0.0 | 1.0 |  | 6.0 |  | 1.0 | 2.1 | 1.2 | 2.4 | 2.2 | 4.0 |  | 1.0 | 4.0 | 2.0 |
| **A*3303** | **16.2** | 7.5 |  |  |  |  |  |  |  |  |  |  |  |  |  |  |
| **A*6801** | **0.3** | 0.0 | 1.0 |  | 4.0 |  |  | 1.4 | 4.7 | 4.2 | 4.4 | 3.0 | 8.0 | 11.0 | 4.0 | 2.0 |
| SUM | **100** | 97 | 76 | 72 | 71 | 69 | 58 | 99 | 96 | 89 | 97 | 85 | 79 | 91 | 57 | 31 |

* Only alleles present in the South Korean populations (in this study) are included. The other population data were reported by Johansson et al [43] and referenced on Allelefrequencies.net.

** From Allelefrequencies.net: Japan pop 16
