# Supplementary material for: Allele and haplotype frequencies of human leukocyte antigen-A, -B, -C, -DRB1, -DRB3/4/5, -DQA1, -DQB1, -DPA1, and -DPB1 by next generation sequencing-based typing in Koreans in South Korea
Source: PLoS One. 2021 Jun 21;16(6):e0253619. doi: 10.1371/journal.pone.0253619 (PMC8216545; doi:10.1371/journal.pone.0253619)
Supplement: S16 Table — (DOCX) [file pone.0253619.s016.docx]

**S16 Table.** HLA-B allele frequencies of 16 populations*

| **alleles** | **South Korean** | **Japanese**** | **Han Chinese** | **Southeast Asian** | **Southwest Asian** | **Oceanian** | **Australian** | **Northern Sami** | **Southern Sami** | **Non-Sami Swedish** | **Finnish** | **European** | **South American** | **North American** | **North African** | **Sub-Saharan African** |
| --- | --- | --- | --- | --- | --- | --- | --- | --- | --- | --- | --- | --- | --- | --- | --- | --- |
| **B*0702** | **4.9** | 5.6 | 5.0 | 1.0 | 3.0 |  | 1.0 | 19.0 | 18.6 | 20.0 | 14.4 | 14.0 | 1.0 | 4.0 | 3.0 | 4.0 |
| **B*0705** | **0.9** | 0.0 |  |  |  |  |  |  |  |  |  |  |  |  |  |  |
| **B*0801** | **0.9** | 0.0 |  |  | 4.0 |  | 1.0 | 4.9 | 7.0 | 8.7 | 8.9 | 12.0 |  | 2.0 | 8.0 | 4.0 |
| **B*1301** | **3.2** | 1.2 | 1.0 | 8.0 |  | 4.0 | 24.0 |  | 1.2 | 1.2 |  |  |  |  |  |  |
| **B*1302** | **4.9** | 0.3 |  |  |  |  |  |  |  |  |  |  |  |  |  |  |
| **B*1401** | **0.6** | 0.0 |  |  |  |  |  |  |  |  |  |  |  |  |  |  |
| **B*1501** | **11.0** | 7.6 | 7.0 | 3.0 |  |  |  | 15.0 | 14.0 | 9.3 | 12.2 | 5.0 | 2.0 | 7.0 |  |  |
| **B*1507** | **1.2** | 0.7 |  |  |  |  |  |  |  |  |  |  |  |  |  |  |
| **B*1511** | **1.5** | 0.9 | 2.0 |  |  |  |  | 0.3 |  |  |  |  |  |  |  |  |
| **B*1517** | **0.3** |  |  |  |  |  |  |  |  |  |  |  |  |  |  |  |
| **B*1518** | **1.2** | 1.5 |  |  |  |  |  |  |  |  |  |  |  |  |  |  |
| **B*1527** | **0.6** | 0.1 |  |  |  |  |  |  |  |  |  |  |  |  |  |  |
| **B*2704** | **0.3** | 0.2 |  |  |  |  |  |  |  |  |  |  |  |  |  |  |
| **B*2705** | **3.2** | 0.1 | 3.0 |  | 1.0 |  |  | 20.0 | 10.5 | 5.6 | 6.1 | 3.0 |  | 10.0 | 1.0 |  |
| **B*3501** | **5.2** | 8.3 | 6.0 | 2.0 | 6.0 | 1.0 |  | 3.5 | 8.1 | 2.6 | 11.7 | 6.0 | 1.0 | 13.0 | 4.0 | 5.0 |
| **B*3503** | **0.3** |  | 1.0 |  | 3.0 |  |  | 0.7 | 0.8 | 0.6 |  | 2.0 |  |  | 1.0 |  |
| **B*3701** | **0.6** | 0.5 | 2.0 |  |  |  |  | 4.2 | 1.6 | 2.0 |  | 2.0 |  |  |  |  |
| **B*3802** | **1.5** | 0.3 |  |  |  |  |  |  |  |  |  |  |  |  |  |  |
| **B*3901** | **0.9** |  | 1.0 | 5.0 |  | 3.0 | 2.0 | 3.5 | 0.4 | 0.6 | 1.1 | 1.0 | 4.0 | 2.0 | 1.0 |  |
| **B*4001** | **3.2** | 5.4 | 4.0 | 16.0 | 1.0 | 15.0 | 9.0 | 14.0 | 10.1 | 9.7 | 9.4 | 5.0 |  | 2.0 | 1.0 |  |
| **B*4002** | **4.3** | 8.0 | 7.0 | 4.0 | 1.0 | 9.0 | 17.0 | 8.4 | 3.9 | 0.6 | 1.7 | 1.0 | 7.0 | 18.0 | 1.0 |  |
| **B*4003** | **0.9** | 0.4 |  |  |  |  |  |  |  |  |  |  |  |  |  |  |
| **B*4006** | **2.0** | 4.8 |  |  |  |  |  |  |  |  |  |  |  |  |  |  |
| **B*4402** | **1.2** | 0.4 | 3.0 |  | 3.0 |  | 1.0 | 3.5 | 10.1 | 13.5 | 4.4 | 11.0 |  | 2.0 | 6.0 | 1.0 |
| **B*4403** | **8.4** | 6.8 | 5.0 | 1.0 | 3.0 | 1.0 |  | 0.4 | 2.4 | 2.6 | 1.7 | 5.0 |  | 2.0 | 10.0 | 4.0 |
| **B*4601** | **6.1** | 4.8 |  |  |  |  |  |  |  |  |  |  |  |  |  |  |
| **B*4801** | **3.8** | 2.9 | 4.0 | 4.0 |  | 6.0 |  | 0.7 |  |  |  |  |  | 8.0 |  |  |
| **B*5101** | **4.9** | 8.9 | 9.0 | 4.0 | 9.0 | 1.0 |  | 1.0 | 3.5 | 6.3 | 5.6 | 5.0 | 2.0 | 10.0 | 4.0 | 2.0 |
| **B*5102** | **1.2** | 0.2 |  |  |  |  |  |  |  |  |  |  |  |  |  |  |
| **B*5201** | **1.5** | 11.1 |  |  |  |  |  |  |  |  |  |  |  |  |  |  |
| **B*5401** | **7.8** | 7.5 |  |  |  |  |  |  |  |  |  |  |  |  |  |  |
| **B*5419** | **0.3** |  |  |  |  |  |  |  |  |  |  |  |  |  |  |  |
| **B*5502** | **1.7** | 2.7 |  |  |  |  |  |  |  |  |  |  |  |  |  |  |
| **B*5507** | **0.3** |  |  |  |  |  |  |  |  |  |  |  |  |  |  |  |
| **B*5601** | **0.6** | 0.9 |  |  |  |  |  |  |  |  |  |  |  |  |  |  |
| **B*5801** | **5.8** | 0.6 |  |  |  |  |  |  |  |  |  |  |  |  |  |  |
| **B*5901** | **2.9** | 2.0 |  |  |  |  |  |  |  |  |  |  |  |  |  |  |
| **B*6701** | **0.3** | 1.2 |  |  |  |  |  |  |  |  |  |  |  |  |  |  |
| SUM | **100** | 95 | 60 | 48 | 34 | 40 | 55 | 99 | 92 | 83 | 77 | 72 | 17 | 80 | 40 | 20 |

* Only alleles present in the South Korean populations (in this study) are included. The other population data were reported by Johansson et al [43] and referenced on Allelefrequencies.net.

** From Allelefrequencies.net: Japan pop 16
