# Supplementary material for: Allele and haplotype frequencies of human leukocyte antigen-A, -B, -C, -DRB1, -DRB3/4/5, -DQA1, -DQB1, -DPA1, and -DPB1 by next generation sequencing-based typing in Koreans in South Korea
Source: PLoS One. 2021 Jun 21;16(6):e0253619. doi: 10.1371/journal.pone.0253619 (PMC8216545; doi:10.1371/journal.pone.0253619)
Supplement: S17 Table — (DOCX) [file pone.0253619.s017.docx]

**S17 Table.** HLA-C allele frequencies of 13 populations*

| **alleles** | **South Korean** | **Japanese**** | **Han Chinese** | **Australian** | **Southeast Asian** | **South Asian** | **West Asian** | **Oceanian** | **European** | **South American** | **North American** | **North African** | **Sub-Saharan African** |
| --- | --- | --- | --- | --- | --- | --- | --- | --- | --- | --- | --- | --- | --- |
| **C*0102** | **20.8** | 17.6 | 19.8 | 19.8 | 16.5 | 2.9 | 1.0 | 20.8 | 3.2 | 8.8 | 4.5 | 0.4 | 0.5 |
| **C*0103** | **0.9** | 0.3 | 0.6 |  | 0.4 |  | 0.2 | 0.1 | 0.0 | 0.0 | 0.0 |  |  |
| **C*0202** | **1.2** | 0.0 | 0.3 | 0.1 | 0.3 | 0.4 | 2.3 | 0.0 | 4.6 | 2.7 | 4.4 | 2.1 | 4.9 |
| **C*0302** | **6.1** | 0.6 | 7.0 | 0.2 | 7.4 | 4.3 | 2.9 | 0.0 | 0.4 | 1.0 | 1.5 | 0.3 | 2.4 |
| **C*0303** | **13.3** | 13.1 | 6.3 | 6.8 | 4.3 | 1.5 | 1.4 | 8.4 | 4.0 | 2.1 | 4.1 | 0.4 | 0.8 |
| **C*0304** | **9.3** | 12.4 | 11.8 | 2.5 | 10.3 | 1.2 | 0.3 | 7.0 | 5.1 | 7.0 | 6.7 | 1.2 | 4.1 |
| **C*0401** | **6.1** | 4.4 | 5.1 | 21.8 | 5.0 | 7.9 | 13.8 | 9.8 | 10.8 | 12.4 | 13.4 | 11.3 | 10.6 |
| **C*0501** | **1.2** | 0.4 | 0.4 | 3.1 | 0.3 | 0.5 | 1.6 | 0.6 | 5.3 | 3.5 | 6.0 | 2.1 | 1.0 |
| **C*0602** | **4.9** | 0.8 | 5.8 | 3.0 | 4.1 | 12.1 | 11.9 | 0.4 | 8.3 | 3.5 | 8.3 | 11.8 | 13.1 |
| **C*0701** | **0.3** | 0.1 | 0.8 | 5.2 | 1.7 | 6.7 | 6.5 | 1.1 | 11.8 | 6.6 | 12.3 | 7.7 | 10.5 |
| **C*0702** | **11.3** | 12.7 | 17.3 | 8.1 | 16.8 | 9.8 | 5.8 | 13.5 | 10.1 | 9.7 | 12.4 | 2.7 | 3.3 |
| **C*0704** | **1.2** | 0.9 | 0.7 | 0.8 | 1.3 | 1.8 | 0.5 | 0.7 | 1.5 | 0.3 | 1.1 | 0.6 | 2.0 |
| **C*0706** | **2.3** |  | 0.1 |  | 0.1 | 0.4 | 0.2 |  | 0.0 | 0.0 | 0.0 | 0.1 | 0.3 |
| **C*0801** | **3.5** | 7.4 | 9.0 | 0.1 | 10.7 | 1.5 | 1.3 | 6.2 | 0.1 | 1.2 | 1.8 |  | 0.4 |
| **C*0802** | **0.6** | 0.0 | 0.2 | 0.8 | 0.1 | 0.3 | 2.9 | 0.4 | 2.1 | 3.4 | 3.3 | 3.5 | 2.9 |
| **C*0803** | **1.7** | 1.4 | 0.4 |  | 0.3 | 0.0 | 0.0 |  | 0.1 | 0.7 | 0.1 |  |  |
| **C*0806** | **0.3** |  | 0.0 |  |  | 0.1 | 0.0 | 0.0 | 0.0 |  | 0.0 |  |  |
| **C*0822** | **0.9** |  |  |  |  |  |  |  |  |  |  |  |  |
| **C*1202** | **1.5** | 11.2 | 2.6 | 0.1 | 3.2 | 6.1 | 3.2 | 3.4 | 1.1 | 0.7 | 1.6 | 1.3 | 0.4 |
| **C*1203** | **0.6** | 0.1 | 1.6 | 1.0 | 1.7 | 3.4 | 5.1 | 2.2 | 6.0 | 3.0 | 3.8 | 4.2 | 1.1 |
| **C*1402** | **3.2** | 6.9 | 4.1 | 0.2 | 3.4 | 3.5 | 1.2 | 0.8 | 1.4 | 0.8 | 1.9 | 1.0 | 0.7 |
| **C*1403** | **5.5** | 6.7 | 0.5 |  | 0.3 | 0.1 | 0.1 | 0.3 | 0.0 | 0.1 | 0.2 | 0.1 | 0.7 |
| **C*1439** | **0.3** |  |  |  |  |  |  |  |  |  |  |  |  |
| **C*1502** | **2.6** | 3.1 | 3.1 | 12.8 | 2.9 | 6.4 | 4.1 | 7.1 | 2.5 | 3.3 | 3.0 | 1.4 | 0.7 |
| **C*1505** | **0.9** | 0.0 | 0.5 | 0.1 | 0.7 | 0.5 | 1.3 | 0.2 | 0.4 | 0.3 | 0.8 | 1.2 | 0.5 |
| SUM | **100** | 99 | 98 | 86 | 91 | 71 | 67 | 83 | 78 | 71 | 91 | 53 | 60 |

* Only alleles present in the South Korean populations (in this study) are included. The other population data were referenced on Allelefrequencies.net.

** From Allelefrequencies.net: Japan pop 16
