# Supplementary material for: Allele and haplotype frequencies of human leukocyte antigen-A, -B, -C, -DRB1, -DRB3/4/5, -DQA1, -DQB1, -DPA1, and -DPB1 by next generation sequencing-based typing in Koreans in South Korea
Source: PLoS One. 2021 Jun 21;16(6):e0253619. doi: 10.1371/journal.pone.0253619 (PMC8216545; doi:10.1371/journal.pone.0253619)
Supplement: S18 Table — (DOCX) [file pone.0253619.s018.docx]

**S18 Table.** HLA-DRB1 allele frequencies of 16 populations*

| **alleles** | **South Korean** | **Japanese**** | **Han Chinese** | **Southeast Asian** | **Southwest Asian** | **Oceanian** | **Australian** | **Northern Sami** | **Southern Sami** | **Non-Sami Swedish** | **Finnish** | **European** | **South American** | **North American** | **North African** | **Sub-Saharan African** |
| --- | --- | --- | --- | --- | --- | --- | --- | --- | --- | --- | --- | --- | --- | --- | --- | --- |
| **DRB1*0101** | **5.8** | 5.8 | 5.7 | 0.1 | 4.1 | 0.2 | 0.7 | 9.5 | 7.6 | 7.5 | 12.8 | 8.0 | 0.3 | 0.6 | 1.0 | 1.4 |
| **DRB1*0301** | **2.3** | 0.4 | 3.6 | 2.8 | 10.9 | 1.5 | 3.2 | 7.2 | 8.3 | 9.3 | 8.9 | 14.3 | 0.3 | 0.9 | 16.1 | 6.5 |
| **DRB1*0401** | **1.2** | 1.1 | 2.8 |  | 1.4 |  | 0.4 | 5.1 | 11.2 | 20.0 | 13.2 | 10.0 |  | 8.9 | 0.2 | 0.5 |
| **DRB1*0403** | **3.5** | 2.8 | 2.2 | 4.4 | 4.7 | 1.1 | 0.7 |  | 0.4 |  |  | 0.3 | 2.4 | 5.3 | 2.6 | 0.1 |
| **DRB1*0404** | **0.6** | 0.3 | 0.9 | 3.5 | 2.0 |  | 1.1 | 1.4 | 7.6 | 3.6 |  | 4.3 | 0.5 | 3.6 | 0.8 | 0.2 |
| **DRB1*0405** | **8.4** | 14.0 | 6.1 | 4.4 | 2.3 | 4.8 | 7.5 | 0.3 | 0.4 |  | 6.2 | 0.3 | 0.5 |  | 4.6 | 1.4 |
| **DRB1*0406** | **5.2** | 2.9 |  |  |  |  |  |  |  |  |  |  |  |  |  |  |
| **DRB1*0407** | **0.3** | 0.6 | 0.4 |  | 0.7 |  |  |  | 0.8 |  |  | 1.4 | 8.8 | 8.5 |  |  |
| **DRB1*0410** | **0.9** | 2.0 |  |  |  |  |  |  |  |  |  |  |  |  |  |  |
| **DRB1*0701** | **7.8** | 0.6 | 8.5 | 1.1 | 8.2 | 1.4 | 2.9 | 0.7 | 5.5 | 9.1 | 4.5 | 14.6 | 1.2 | 0.8 | 15.3 | 5.3 |
| **DRB1*0802** | **2.6** | 4.3 | 2.6 | 0.1 | 0.8 |  |  | 2.9 | 1.2 |  |  | 0.1 | 7.6 | 12.0 |  | 0.2 |
| **DRB1*0803** | **11.6** | 7.8 | 3.9 | 10.6 | 1.0 | 3.4 | 32.5 | 0.3 |  |  |  | 0.2 |  |  | 0.3 | 0.1 |
| **DRB1*0901** | **5.5** | 13.2 | 9.1 | 7.9 | 1.3 | 1.4 |  | 16.4 | 8.3 | 1.8 | 0.6 | 0.6 | 6.9 | 3.7 | 1.0 | 1.1 |
| **DRB1*1001** | **0.6** | 0.4 | 2.1 | 0.9 | 5.9 |  | 0.4 | 3.0 | 2.8 | 1.0 | 1.7 | 0.7 |  |  | 3.4 | 3.5 |
| **DRB1*1101** | **4.1** | 2.6 | 3.5 | 10.1 | 8.3 | 14.8 |  | 11.6 | 5.1 | 7.1 | 2.0 | 2.9 | 0.2 | 4.4 | 5.4 | 11.0 |
| **DRB1*1145** | **0.3** |  |  |  |  |  |  |  |  |  |  |  |  |  |  |  |
| **DRB1*1201** | **4.9** | 3.7 | 6.2 | 1.9 | 1.6 | 2.3 | 1.4 | 1.7 | 2.4 | 1.4 | 1.7 | 1.3 |  | 0.2 | 0.4 | 3.5 |
| **DRB1*1202** | **3.5** | 1.8 |  |  |  |  |  |  |  |  |  |  |  |  |  |  |
| **DRB1*1301** | **1.2** | 0.7 | 3.7 | 0.3 | 7.2 | 0.2 | 1.4 | 5.1 | 9.3 | 3.0 | 18.0 | 4.5 | 0.7 | 0.2 | 3.0 | 5.0 |
| **DRB1*1302** | **9.3** | 5.9 | 6.3 | 0.8 | 3.8 | 0.6 |  | 2.8 | 4.8 | 4.6 | 1.3 | 3.1 |  | 0.2 | 7.6 | 10.1 |
| **DRB1*13198** | **0.3** |  |  |  |  |  |  |  |  |  |  |  |  |  |  |  |
| **DRB1*1403** | **0.9** | 0.8 |  |  |  |  |  |  |  |  |  |  |  |  |  |  |
| **DRB1*1404** | **0.3** | 0.0 |  |  |  |  |  |  |  |  |  |  |  |  |  |  |
| **DRB1*1405** | **3.8** | 2.2 |  |  |  |  |  |  |  |  |  |  |  |  |  |  |
| **DRB1*1406** | **0.9** | 1.3 |  |  |  |  |  |  |  |  |  |  |  |  |  |  |
| **DRB1*1407** | **0.6** | 0.1 |  |  |  |  |  |  |  |  |  |  |  |  |  |  |
| **DRB1*1454** | **3.5** | 1.4 |  |  |  |  |  |  |  |  |  |  |  |  |  |  |
| **DRB1*14142** | **0.3** |  |  |  |  |  |  |  |  |  |  |  |  |  |  |  |
| **DRB1*1501** | **6.7** | 8.1 | 9.9 | 5.2 | 6.5 | 7.0 | 3.2 | 7.5 | 14.7 | 20.2 | 14.4 | 16.2 |  | 0.8 | 7.9 | 0.8 |
| **DRB1*1502** | **3.5** | 10.1 |  |  |  |  |  |  |  |  |  |  |  |  |  |  |
| **DRB1*1602** | **0.3** | 0.8 |  |  |  |  |  |  |  |  |  |  |  |  |  |  |
| SUM | **100** | 95 | 77 | 54 | 70 | 38 | 55 | 75 | 90 | 88 | 85 | 82 | 29 | 50 | 69 | 50 |

* Only alleles present in the South Korean populations (in this study) are included. The other population data were reported by Johansson et al [43] and referenced on Allelefrequencies.net.
