# Supplementary material for: Allele and haplotype frequencies of human leukocyte antigen-A, -B, -C, -DRB1, -DRB3/4/5, -DQA1, -DQB1, -DPA1, and -DPB1 by next generation sequencing-based typing in Koreans in South Korea
Source: PLoS One. 2021 Jun 21;16(6):e0253619. doi: 10.1371/journal.pone.0253619 (PMC8216545; doi:10.1371/journal.pone.0253619)
Supplement: S19 Table — (DOCX) [file pone.0253619.s019.docx]

**S19 Table.** HLA-DQA1 allele frequencies of 15 populations*

| **alleles** | **South Korean** | **Japanese**** | **Han Chinese** | **Southeast Asian***** | **Southwest Asian** | **Oceanian** | **Australian** | **Northern Sami** | **Southern Sami** | **Non-Sami Swedish** | **European** | **South American** | **North American** | **North African** | **Sub-Saharan African** |
| --- | --- | --- | --- | --- | --- | --- | --- | --- | --- | --- | --- | --- | --- | --- | --- |
| **DQA1*0101** | **6.7** | 6.6 | 7.6 | 5.1 | 14.5 | 23.4 | 14.3 | 13.5 | 13.6 | 10.3 | 10.5 |  | 1.1 | 11.8 | 16.0 |
| **DQA1*0102** | **16.2** | 13.4 | 20.2 | 19.5 | 16.5 | 45.8 | 11.1 | 10.4 | 19.4 | 25.6 | 21.2 |  | 1.1 | 18.4 | 31.9 |
| **DQA1*0103** | **14.2** | 19.2 | 11.1 | 11.0 | 9.0 | 3.9 | 33.6 | 5.3 | 8.8 | 3.0 | 8.3 | 1.2 | 0.2 | 3.6 | 5.8 |
| **DQA1*0104** | **7.8** | 4.7 | 6.3 |  |  |  |  |  |  |  |  |  |  |  |  |
| **DQA1*0105** | **0.6** | 0.6 | 1.2 |  |  |  |  |  |  |  |  |  |  |  |  |
| **DQA1*0201** | **7.8** | 0.4 | 5.6 | 10.2 | 8.8 | 1.7 | 2.9 | 0.7 | 5.4 | 9.1 | 13.9 | 1.2 | 1.2 | 16.9 | 5.4 |
| **DQA1*0301** | **11.3** | 11.0 | 9.7 | 28.8 |  | 10.3 | 19.6 | 5.6 | 13.6 | 16.5 | 7.8 | 27.4 | 46.7 | 16.1 | 5.8 |
| **DQA1*0302** | **5.5** | 14.4 | 9.7 |  |  |  |  | 20.1 | 15.1 | 10.3 | 2.9 |  | 5.0 |  |  |
| **DQA1*0303** | **11.0** | 16.6 | 11.3 |  |  |  |  |  |  |  |  |  |  |  |  |
| **DQA1*0401** | **0.9** | 2.8 | 1.2 | 0.8 | 2.5 |  |  | 22.9 | 7.4 | 6.7 | 3.2 | 18.8 | 11.3 | 4.1 | 12.9 |
| **DQA1*0501** | **3.2** | 0.1 | 3.1 | 17.8 | 32.9 | 9.9 | 18.2 | 21.2 | 16.7 | 18.5 | 27.3 | 51.5 | 32.0 | 28.9 | 21.9 |
| **DQA1*0503** | **1.5** | 2.8 | 2.8 |  |  |  |  |  |  |  |  |  |  |  |  |
| **DQA1*0505** | **4.6** | 4.4 | 6.8 |  |  |  |  |  |  |  |  |  |  |  |  |
| **DQA1*0506** | **1.2** | 0.3 |  |  |  |  |  |  |  |  |  |  |  |  |  |
| **DQA1*0507** | **0.6** |  |  |  |  |  |  |  |  |  |  |  |  |  |  |
| **DQA1*0508** | **2.0** | 0.8 |  |  |  |  |  |  |  |  |  |  |  |  |  |
| **DQA1*0601** | **5.2** | 2.0 | 3.4 | 6.8 | 0.8 | 5.2 | 0.4 | 0.3 |  |  | 0.5 |  |  | 0.3 |  |
| SUM | **100** | 100 | 100 | 100 | 85 | 100 | 100 | 100 | 100 | 100 | 95 | 100 | 98 | 100 | 99 |

* Only alleles present in the South Korean populations (in this study) are included. The other population data were reported by Johansson et al [43] and referenced on Allelefrequencies.net.

** From Allelefrequencies.net: Japan pop 16

*** From Allelefrequencies.net: China Urumqi Han
