# Supplementary material for: Allele and haplotype frequencies of human leukocyte antigen-A, -B, -C, -DRB1, -DRB3/4/5, -DQA1, -DQB1, -DPA1, and -DPB1 by next generation sequencing-based typing in Koreans in South Korea
Source: PLoS One. 2021 Jun 21;16(6):e0253619. doi: 10.1371/journal.pone.0253619 (PMC8216545; doi:10.1371/journal.pone.0253619)
Supplement: S20 Table — (DOCX) [file pone.0253619.s020.docx]

**S20 Table.** HLA-DQB1 allele frequencies of 16 populations*

| **alleles** | **South Korean** | **Japanese**** | **Han Chinese** | **Southeast Asian** | **Southwest Asian** | **Oceanian** | **Australian** | **Northern Sami** | **Southern Sami** | **Non-Sami Swedish** | **Finnish** | **European** | **South American** | **North American** | **North African** | **Sub-Saharan African** |
| --- | --- | --- | --- | --- | --- | --- | --- | --- | --- | --- | --- | --- | --- | --- | --- | --- |
| **DQB1*0201** | **2.3** | 0.2 | 2.9 | 9.3 | 16.3 | 3.0 | 5.0 | 7.4 | 8.1 | 9.9 | 14.3 | 18.7 | 1.7 | 2.0 | 34.1 | 15.2 |
| **DQB1*0202** | **7.5** | 0.3 | 5.4 |  |  |  |  | 0.3 | 2.6 | 6.2 | 2.9 | 1.0 |  |  |  | 0.1 |
| **DQB1*0301** | **16.5** | 11.2 | 12.8 | 26.7 | 25.7 | 23.7 | 7.9 | 17.0 | 15.1 | 18.1 | 6.0 | 17.8 | 51.9 | 42.7 | 15.9 | 17.4 |
| **DQB1*0302** | **11.9** | 10.8 | 10.2 | 3.2 | 12.7 | 1.6 | 3.2 | 5.9 | 14.0 | 16.1 | 21.8 | 10.2 | 23.1 | 31.7 | 8.9 | 1.6 |
| **DQB1*0303** | **6.1** | 15.0 | 10.5 | 11.4 | 1.8 | 1.9 | 1.1 | 17.0 | 10.9 | 4.8 | 7.9 | 4.3 | 6.4 | 4.1 | 1.0 | 0.8 |
| **DQB1*0313** | **0.3** |  |  |  |  |  |  |  |  |  |  |  |  |  |  |  |
| **DQB1*0401** | **8.4** | 13.7 | 7.7 |  |  |  |  |  |  |  |  |  |  |  |  |  |
| **DQB1*0402** | **1.7** | 4.4 | 3.9 |  | 1.6 | 1.8 | 21.8 | 22.9 | 7.4 | 6.7 | 7.1 | 3.3 | 16.4 | 14.8 | 3.9 | 8.7 |
| **DQB1*0501** | **6.7** | 7.1 | 8.8 | 10.9 | 8.4 | 8.5 | 2.1 | 12.9 | 11.6 | 8.5 | 11.4 | 10.8 | 0.5 | 0.7 | 10.2 | 19.0 |
| **DQB1*0502** | **3.5** | 2.3 | 5.2 |  |  |  |  |  |  |  |  |  |  |  |  |  |
| **DQB1*0503** | **5.2** | 3.2 | 3.1 | 1.9 | 5.5 | 10.7 | 20.4 | 0.7 | 1.9 | 1.8 |  | 3.1 |  | 2.8 | 2.4 | 0.6 |
| **DQB1*0601** | **13.3** | 18.8 | 10.3 |  |  |  |  |  |  |  |  |  |  |  |  |  |
| **DQB1*0602** | **6.4** | 6.3 | 7.4 | 2.3 | 6.7 | 6.2 | 2.9 | 7.6 | 13.2 | 20.4 | 18.1 | 10.7 |  | 0.8 | 8.5 | 21.4 |
| **DQB1*0603** | **1.2** | 0.5 | 1.1 | 0.6 | 3.9 | 0.2 | 1.8 | 5.2 | 10.1 | 2.8 | 7.5 | 8.2 |  | 0.2 | 2.0 | 2.8 |
| **DQB1*0604** | **6.1** | 5.9 | 6.3 | 0.4 | 3.7 | 0.1 |  | 2.8 | 4.7 | 4.6 | 1.5 | 1.9 |  |  | 4.9 | 6.4 |
| **DQB1*0609** | **3.2** | 0.5 |  |  |  |  |  |  |  |  |  |  |  |  |  |  |
| SUM | **100** | 99 | 95 | 66 | 86 | 57 | 66 | 99 | 99 | 99 | 98 | 90 | 100 | 99 | 91 | 94 |

* Only alleles present in the South Korean populations (in this study) are included. The other population data were reported by Johansson et al [43] and referenced on Allelefrequencies.net.

** From Allelefrequencies.net: Japan pop 16
