# Supplementary material for: Allele and haplotype frequencies of human leukocyte antigen-A, -B, -C, -DRB1, -DRB3/4/5, -DQA1, -DQB1, -DPA1, and -DPB1 by next generation sequencing-based typing in Koreans in South Korea
Source: PLoS One. 2021 Jun 21;16(6):e0253619. doi: 10.1371/journal.pone.0253619 (PMC8216545; doi:10.1371/journal.pone.0253619)
Supplement: S21 Table — (DOCX) [file pone.0253619.s021.docx]

**S21 Table.** HLA-DPA1 allele frequencies of 10 populations*

| **alleles** | **South Korean** | **Japanese**** | **Southeast Asian** | **South Asian***** | **Oceanian** | **European** | **Brazilian****** | **South American** | **North American** | **Sub-Saharan African** |
| --- | --- | --- | --- | --- | --- | --- | --- | --- | --- | --- |
| **DPA1*0103** | **43.9** | 40.3 | 32.6 | 59.3 | 41.9 | 81.6 | 71.7 | 17.4 | 91.6 | 31.4 |
| **DPA1*0104** | **0.3** | 0.0 | 0.1 | 2.5 |  | 0.2 |  | 0.2 |  | 0.3 |
| **DPA1*0201** | **15.6** | 16.0 | 10.8 |  |  | 7.5 |  | 14.9 | 2.6 | 3.7 |
| **DPA1*0202** | **40.2** | 43.5 | 50.1 |  | 53.0 | 0.7 |  |  | 3.9 | 2.4 |
| SUM | **100** | 99 | 93 | 61 | 94 | 90 | 71 | 32 | 98 | 37 |

* Only alleles present in the South Korean populations (in this study) are included. The other population data were referenced on Allelefrequencies.net.

** From Allelefrequencies.net: Japan pop 16

*** From Allelefrequencies.net: India bombay

**** From Allelefrequencies.net: Brazil Ticuna
