# Supplementary material for: Allele and haplotype frequencies of human leukocyte antigen-A, -B, -C, -DRB1, -DRB3/4/5, -DQA1, -DQB1, -DPA1, and -DPB1 by next generation sequencing-based typing in Koreans in South Korea
Source: PLoS One. 2021 Jun 21;16(6):e0253619. doi: 10.1371/journal.pone.0253619 (PMC8216545; doi:10.1371/journal.pone.0253619)
Supplement: S22 Table — (DOCX) [file pone.0253619.s022.docx]

**S22 Table.** HLA-DPB1 allele frequencies of 14 populations*

| **alleles** | **South Korean** | **Japanese**** | **Han Chinese** | **Southeast Asian** | **South Asian** | **West Asian** | **Australian** | **Oceanian** | **European** | **South American** | **North American** | **North African** | **Sub-Saharan African** |
| --- | --- | --- | --- | --- | --- | --- | --- | --- | --- | --- | --- | --- | --- |
| **DPB1*0201** | **25.1** | 23.8 | 14.5 | 4.0 | 17.9 |  | 13.8 | 8.5 | 15.1 | 7.4 | 11.0 | 25.5 | 9.2 |
| **DPB1*0202** | **5.2** | 3.9 | 7.5 | 2.9 | 0.1 |  |  | 0.5 | 0.8 | 0.4 | 0.6 | 1.3 | 0.1 |
| **DPB1*0301** | **4.6** | 5.1 | 4.5 | 1.6 | 4.4 | 6.7 | 2.2 | 1.6 | 14.5 | 5.2 | 6.4 | 10.3 | 4.5 |
| **DPB1*0401** | **6.1** | 5.0 | 10.4 | 4.1 | 27.1 | 36.0 | 10.4 | 17.3 | 38.2 | 12.0 | 26.0 | 23.8 | 5.2 |
| **DPB1*0402** | **8.7** | 9.8 | 4.4 | 2.2 | 4.5 | 20.1 |  | 3.2 | 10.6 | 41.1 | 29.6 | 11.0 | 21.3 |
| **DPB1*0501** | **34.1** | 37.4 | 36.8 | 18.6 | 0.6 |  | 51.9 | 44.4 | 1.9 | 2.4 | 4.9 | 0.2 | 0.1 |
| **DPB1*0901** | **3.2** | 10.3 | 1.0 | 0.4 | 4.3 | 0.9 |  | 0.1 | 1.3 | 0.4 | 0.6 | 0.9 | 0.2 |
| **DPB1*1301** | **5.8** | 1.8 | 5.7 | 4.4 | 7.6 | 1.7 | 0.7 | 3.1 | 1.8 | 3.8 | 2.3 | 1.7 | 4.1 |
| **DPB1*1401** | **2.3** | 1.3 | 3.0 | 1.7 | 4.0 | 3.0 |  | 1.1 | 1.7 | 14.9 | 1.3 | 1.3 | 0.4 |
| **DPB1*1501** | **0.3** |  |  | 0.1 | 1.1 | 1.0 | 1.9 |  | 0.7 | 0.1 | 0.6 | 2.5 | 0.8 |
| **DPB1*1701** | **3.2** | 0.3 | 3.3 | 0.7 | 1.3 | 2.9 |  | 0.1 | 1.3 | 1.4 | 1.7 | 8.3 | 7.2 |
| **DPB1*3601** | **0.3** | 0.0 |  | 0.1 |  |  |  |  |  |  | 0.0 |  |  |
| **DPB1*3801** | **0.3** | 0.1 | 0.2 | 0.1 |  |  |  |  |  |  | 0.0 |  |  |
| **DPB1*10401** | **0.3** |  | 0.1 | 0.1 |  |  |  |  |  |  | 0.0 |  |  |
| **DPB1*13501** | **0.3** |  |  | 1.4 |  |  |  |  |  |  |  |  |  |
| **DPB1*41401** | **0.3** |  |  | 0.0 |  |  |  |  |  |  |  |  |  |
| SUM | **100** | 98 | 91 | 42 | 72 | 72 | 80 | 79 | 87 | 89 | 85 | 86 | 53 |

* Only alleles present in the South Korean populations (in this study) are included. The other population data were referenced on Allelefrequencies.net.

** From Allelefrequencies.net: Japan pop 16
